# Morphological Continua Make Poor Species: Genus-Wide Morphometric Survey of the European Bee Orchids (*Ophrys* L.)

**DOI:** 10.3390/biology12010136

**Published:** 2023-01-16

**Authors:** Richard M. Bateman, Paula J. Rudall

**Affiliations:** Jodrell Laboratory, Royal Botanic Gardens Kew, Richmond, Surrey TW9 3DS, UK

**Keywords:** demographic systematics, ethology, evolution, morphometrics, natural selection, next-generation sequencing, ordination, phylogeny, reproductive isolation, sexual deceit, speciation, species circumscription

## Abstract

**Simple Summary:**

Our frequently deployed approach to optimally circumscribing species requires large-scale field sampling within and between populations for large numbers of morphometric characters, followed by multivariate ordinations to objectively seek discontinuities (or, failing that, zones of limited overlap) among sets of populations considered to represent species. Corresponding boundaries are sought in DNA-based outputs, either phylogenies or preferably ordinations based on population genetic data. Herein, we analyse within a molecular phylogenetic framework detailed morphometric data for the charismatic bee orchids (*Ophrys*), seeking a ‘mesospecies’ species concept that might provide a compromise between the nine ‘macrospecies’ recognised primarily through DNA barcoding and the several hundred ‘microspecies’ recognised primarily through perceived pollinator specificity. Our analyses failed to find robust groupings that could be regarded as credible mesospecies, instead implying that each macrospecies constitutes a morphological continuum. This problematic result encouraged us to reappraise both our morphometric approach and the relative merits of the contrasting macrospecies and microspecies concepts, and to reiterate the key role played by genetics in species circumscription.

**Abstract:**

Despite (or perhaps because of) intensive multidisciplinary research, opinions on the optimal number of species recognised within the Eurasian orchid genus *Ophrys* range from nine to at least 400. The lower figure of nine macrospecies is based primarily on seeking small but reliable discontinuities in DNA ‘barcode’ regions, an approach subsequently reinforced and finessed via high-throughput sequencing studies. The upper figure of ca. 400 microspecies reflects the morphological authoritarianism of traditional taxonomy combined with belief in extreme pollinator specificity caused by reliance on pollination through pseudo-copulation, enacted by bees and wasps. Groupings of microspecies that are less inclusive than macrospecies are termed mesospecies. Herein, we present multivariate morphometric analyses based on 51 characters scored for 457 individual plants that together span the full morphological and molecular diversity within the genus *Ophrys*, encompassing 113 named microspecies that collectively represent all 29 mesospecies and all nine macrospecies. We critique our preferred morphometric approach of accumulating heterogeneous data and analysing them primarily using principal coordinates, noting that our conclusions would have been strengthened by even greater sampling and the inclusion of data describing pseudo-pheromone cocktails. Morphological variation within *Ophrys* proved to be exceptionally multidimensional, lacking strong directional trends. Multivariate clustering of plants according to prior taxonomy was typically weak, irrespective of whether it was assessed at the level of macrospecies, mesospecies or microspecies; considerable morphological overlap was evident even between subsets of the molecularly differentiable macrospecies. Characters supporting genuine taxonomic distinctions were often sufficiently subtle that they were masked by greater and more positively correlated variation that reflected strong contrasts in flower size, tepal colour or, less often, plant size. Individual macrospecies appear to represent morphological continua, within which taxonomic divisions are likely to prove arbitrary if based exclusively on morphological criteria and adequately sampled across their geographic range. It remains unclear how much of the mosaic of subtle character variation among the microspecies reflects genetic versus epigenetic or non-genetic influences and what proportion of any contrasts observed in gene frequencies can be attributed to the adaptive microevolution that is widely considered to dictate speciation in the genus. Moreover, supplementing weak morphological criteria with extrinsic criteria, typically by imposing constraints on geographic location and/or supposed pollinator preference, assumes rather than demonstrates the presence of even the weakest of species boundaries. Overall, it is clear that entities in *Ophrys* below the level of macrospecies have insufficiently structured variation, either phenotypic or genotypic, to be resolved into discrete, self-circumscribing (“natural”) entities that can legitimately be equated with species as delimited within other less specialised plant genera. Our search for a non-arbitrary (meso)species concept competent to circumscribe an intermediate number of species has so far proven unsuccessful.

## 1. Introduction

### 1.1. Bee Orchids as an Evolutionary Case-Study

Few plant genera have gained as much academic attention as *Ophrys* L. (bee orchids). When attracting researchers, orchids per se have the advantage of being renowned for their complexity and diversity, as well as being readily recognisable (they are almost unique in developing their ‘male’ and ‘female’ reproductive organs as a single unified structure, the gynostemium [1,2,3]). But even among orchid genera, bee orchids are exceptionally charismatic, largely as a result of their near-ubiquitous reliance on pollination through sexual deception—specifically, through attempts made by (typically) bees and wasps to mate with their intricate flowers. Passing insects are attracted by first biochemical, then visual and finally tactile cues. Their combined effect must be sufficiently convincing to trick a male insect into twice believing that he is interacting with a conspecific female—collecting at least one of the two pollinaria from one flower and soon depositing it on the stigma of another flower. Pollination via pseudo-copulation is a high-risk strategy that achieves considerably less success than deceitfully promising and far less success than actually providing, a nectar reward [4,5,6]. Although pseudo-copulatory pollination is gradually being detected in other groups of plants, both within and outwith the orchid family, *Ophrys* remains the most intensively researched system of sexual deception and therefore acts as the archetypal textbook case-study [7,8].

Initial studies of evolution within the genus focused on behavioural ecology and functional morphology, therefore concentrating on visual and tactile cues [9,10,11]. Even among orchids, the labellum (the median petal, evolutionarily modified to act as a combined landing stage and sex-doll) is exceptionally complex in its overall three-dimensional topography, outline, markings and in the distribution of trichomes, papillae and glandular cells across its surface [12,13,14]. Technological advances enabled later studies to reveal biochemical aspects of the flower’s phenotype to be equally extraordinary [15,16,17,18]. Complex cocktails of volatile exudates were shown to function as reputedly species-specific pseudo-pheromones, constituting the earliest-acting and arguably the most important of the three categories of pollinator cues. Early genetic work focused on determining relationships among taxa within the genus [19,20,21], but more recently, far larger datasets have begun to allow the identification of genes responsible for some of these intriguing phenotypic features [18,22,23].

Unsurprisingly, *Ophrys* has become a textbook case of what are widely regarded as numerous accumulated adaptations aiding pseudo-copulation, assumed by most authors to be largely the product of speciation through natural selection. In the eyes of many observers, *Ophrys* represents a classic example of recent, rapid and ongoing adaptive radiation, a position that, by definition, requires an exceptionally high speciation rate [24,25,26]. However, this undeniably attractive interpretation is valid only if the many supposed species that are the end-product of the radiation are genuine, rather than the consequence of a seriously flawed species concept that gives greater emphasis to evolutionary process than evolutionary product [27,28,29].

### 1.2. The Explicit Taxonomic Controversy: Microspecies, Mesospecies and Macrospecies

As well as becoming a classic case-study of pseudo-copulatory pollination and adaptive radiation, the genus *Ophrys* has also become a classic case-study of taxonomic controversy—a controversy of sufficient gravitas that it has now extended beyond the realm of systematic biology [27,30], even attracting the attention of philosophers of science [31]. In contrast with today, for much of the 20th century, the taxonomy of *Ophrys* was remarkably stable. Pursued through species concepts that were rooted in perceived (rather than quantified) degrees of morphological difference, several authoritative (but also inevitably authoritarian) classifications were published between 1960 and 1990 that recognised between 19 and 21 species, most of them congruent among these studies, together with a larger number of subspecies [32,33,34]. That traditional view of relatively few species plus many subspecies extended into some 21st century treatments; for example, the *Ophrys* monograph by Pedersen and Faurholdt [35] listed just 19 species and gave rise a decade later to a European orchid flora that listed only 22 species [36].

However, the early 1990s witnessed the first of two radical revolutions that impacted heavily on the taxonomy of *Ophrys*. Two 1994 publications elevated to species level many taxa that had been regarded by previous authors as subspecies or varieties: the technical monograph of Devillers and Devillers-Terschuren [37] listed 150 species, and the first edition of Delforge’s European orchid flora recognised 142 species, a figure that presaged linear increases through three subsequent editions to 215, 252 and 353 species, respectively [38,39]. Many of these putative species were credited with only very restricted geographic distributions. We contend that this order-of-magnitude increase in recognised species was driven largely by an increasingly widely held belief that the unusual pseudo-copulatory pollination mechanism of *Ophrys* typically yielded a single preferred (or even sole) insect pollinator for each orchid species. This assumption led logically to the dubious conclusion that whatever phenotypic differences were apparent among taxa should be considered sufficient to justify their status as different species [40,41,42]. This concept yielded an increasing number of supposed local endemics, a mind-set that in turn encouraged recognition as different species of morphologically similar populations inhabiting contrasting geographic locations (it is no accident that *Ophrys* biodiversity is judged to peak among the numerous islands of the Aegean [25]). The species recognised in these high-diversity classifications were later termed ‘**microspecies**’ by Bateman [28,29,43].

**Figure 1:**
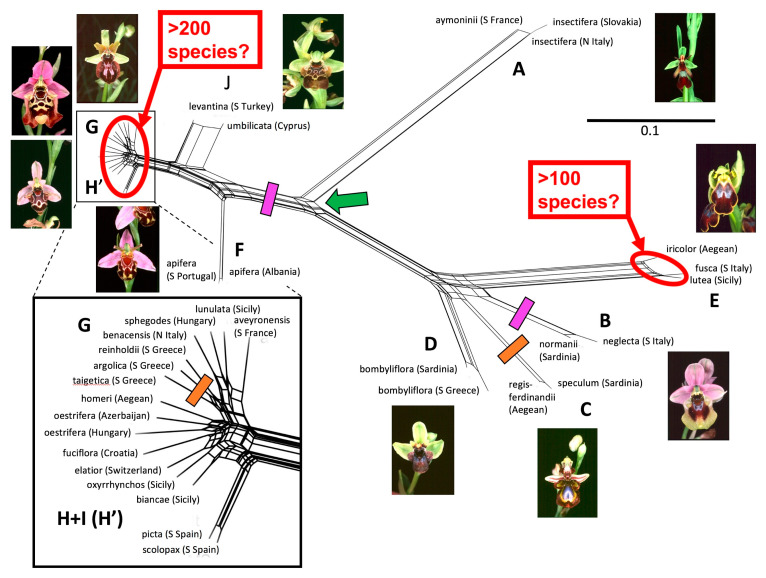
Unrooted *SplitsTree* network based on 4059 RAD-seq-derived single nucleotide polymorphisms (SNPs) for 32 plants that together represent the nine *Ophrys* macrospecies (**A**–**H’**,**J**) circumscribed by Bateman et al. [43]. Inset: Magnified view of that portion of the topology that represents groups G and H’. The root of the tree remains equivocal but certainly lies close to the green-arrowed node. Red ovals emphasise the poverty of genetic variation present in the microspecies-rich groups E, G and H’. Pink bars indicate two predicted origins of the ability to infuse sepals and petals with pink–purple anthocyanin pigments; brown bars indicate two predicted origins of the ability to generate longitudinal stripes of red–brown pigment on the lateral sepals.

The second taxonomic revolution began in 2008, when Devey et al. [21] (see also [28,44]) applied DNA barcoding techniques (nuclear ribosomal ITS plus two plastid regions) to a wide range of named *Ophrys* taxa but were able to recognise with reasonable confidence only ten groupings (labelled A–J). Moreover, many of the individual plants analysed yielded multiple ITS ribotypes, implying recent and most probably ongoing gene-flow among microspecies. The subsequent application of next-generation sequencing techniques to a more restricted range of microspecies confirmed earlier DNA ‘barcode’ results but further reduced the number of groupings that could reliably be recognised to just nine (Figure 1). These nine essentially self-circumscribing (‘natural’) taxonomic units were termed ‘**macrospecies**’ by Bateman [28,29,43,45].

Many observers (including, we admit, ourselves) felt intuitively that the genus *Ophrys* should contain more than nine species but considerably fewer than 353 species. Such intermediate classifications have persisted into the 21st century. At the time of writing (November 2022), the authoritarian (but by no means authoritative) *World Checklist of Selected Plant Families* (WCSP) lists an impressive 2356 formal epithets (including those of hybrid ‘nothospecies’) for the genus *Ophrys* but nonetheless conservatively recognises only 29 embarrassingly heterogeneous species. Both Kreutz [46] and Baumann et al. [47] listed 65 species plus roughly three times that number of subspecies, thereby encompassing approximately as many formal epithets as did the exclusively microspecies-based classification of Delforge [39]. However, Delforge [39] did usefully organise his 353 microspecies into 29 intermediate groups. Herein, those supraspecific groups of Delforge, together with the species listed in any other classification of *Ophrys* that recognises between 20 and 65 species, are termed ‘**mesospecies**’, again following Bateman [28,29,43,45]. The mesospecies is the most heterogeneous of the three categories of putative species; indeed, it is unclear whether any of the available classifications that includes mesospecies [37,38,39] was constructed on an explicit underlying species concept. Thus, a crucial outstanding question is whether a credible example of such a concept can be developed.

### 1.3. The General Taxonomic Controversy: Natural Versus Artificial Species

The extensive literature on *Ophrys* systematics is matched by that discussing the theory and philosophy of species concepts [26,27,28,29,31,48,49,50,51,52,53,54,55,56,57]. Most philosophically informed taxonomic debates are initially couched in terms of “artificial” versus “natural” species. Members of the various “natural” schools believe that species are fundamental biological entities made cohesive by shared biological processes. Most subscribe to “*the* biological species concept” (harkening back to Ernst Mayr’s [48,49] oft-repeated belief that “species are groups of actually or potentially interbreeding populations which are reproductively isolated from other such groups”). This regrettable but persistent use ignores at least a dozen competing biological species concepts (reviewed by [50,51]). Members of the competing “artificial” schools fall into two broad classes—idealistic and pragmatic. Idealistic members have seriously considered and then rejected the idea that species have independent evolutionary fates, whereas pragmatic members argue that simply creating convenient pigeon-holes for organisms is the most rapid and effective approach to the urgent practical task of categorising the Earth’s biodiversity.

Unfortunately, such elevated philosophical debates tend to ignore the reality of current taxonomic practice, at least as applied to higher plants. Most formal species-level epithets are rooted in the floras, monographs and taxonomic notes generated through 270 years of traditional herbarium taxonomy. Even today, such outputs rarely make any mention of which species concept is being applied, while also routinely side-stepping the fact that science, by definition, requires the formulation of explicit questions that can subsequently be addressed through the gathering and quantitative analysis of appropriate data. In the consistent absence of such cycles of analysis, the species thus circumscribed are, by definition, artificial. Those among the traditional practitioners who take seriously the idea that species should have biological reality hope that their output of morphologically circumscribed alpha-taxonomic species will in the future be proven to be what is euphemistically termed “natural” (a term now so often abused in a taxonomic framework that it arguably no longer has meaning). The most obvious way to test their species hypotheses is through the acquisition of additional categories of data, permitting reciprocal illumination through potential congruence among data-sets.

Returning to the three categories of *Ophrys* species established in Section 1.3—microspecies, mesospecies and macrospecies—are they best viewed as natural or artificial? The strength of the nine macrospecies is that they are unequivocally natural, irrespective of which of the many definitions of “natural” is applied. Their genetic profiles demonstrate clearly that they are bona fide clades that have enjoyed independent evolutionary fates for considerable periods of time, and they are also widely regarded as being readily distinguished through their morphologies. Our use of the term “self-circumscribing” was criticised by reviewers of this paper, but we stand by the concept; members of the same plant species “recognise” each other (albeit unconsciously) irrespective of whether the boundaries that they perceive match those ascribed to them by taxonomists.

In contrast, two radically different taxonomic world-views have combined to generate the current plethora of *Ophrys* microspecies. Although classifications containing hundreds of *Ophrys* species are a recent phenomenon, many of the formal epithets were coined in earlier eras that preceded not only molecular systematics but also Mayr’s biological species concept and the recognition of universal pseudo-copulatory pollination among bee orchids. When first established, the epithets were at that stage undeniably artificial, but it could be argued that their artificiality was recognised by deploying most of them at infraspecific levels. It is only recently that belief in extreme pollinator specificity within *Ophrys* appears to have biologically legitimised the subsequent elevation of these epithets to species level, even though such taxonomic changes are still often performed in the absence of any explicit data analysis. In other cases, it is explicit studies of pollination that prompt circumscription of new *Ophrys* microspecies bearing novel epithets. Thus, *Ophrys* microspecies are hybrid progeny, generated when traditional artificial taxa were reappraised via taxonomic concepts that are rooted explicitly in reproductive biology and could therefore be regarded as strongly natural.

Similar ambiguity inevitably pervades the search for a mesospecies concept that would yield a number of species intermediate to those of the current macrospecies and microspecies concepts. Herein, we have used as our initial framework the 29 mesospecies of Delforge [39], which we suspect primarily reflect pragmatic motives. Although unsupported by a conceptual definition, they offer the great advantage of having an explicit relationship with the 363 microspecies recognised by an experienced European orchidologist. Our present search is focused on finding mesospecies that are both “natural” and can be accommodated within a single conceptual definition of species.

### 1.4. Aims of the Present Study

When preparing to write this paper, we first surveyed 55 papers that (a) compared *Ophrys* taxa, (b) were published during the last quarter-century and (c) presented at least some quantitative data. Among these papers, only 20% included analyses of morphological data, compared for example with 35% that quantified the composition of pseudo-pheromone cocktails. The majority of these data-sets were subjected to various forms of multivariate ordination, but in most cases, the data gathered were confined to sampling between two and five microspecies, typically putatively closely related within a single macrospecies; also, in most cases, only a few morphological characters considered a priori to be of particular significance (termed ‘traits’) were scored [22,58,59,60,61,62,63,64,65]. Consequently, these highly focused studies could only explore the credibility of supposed boundaries separating microspecies; higher-level mesospecies and macrospecies were not tested. The exception to this rule was our own recent morphometric ordination of 124 plants collectively representing 33 microspecies within macrospecies Sphegodes, which primarily sought mesospecies boundaries, employing modern approaches to analysing genotype as well as phenotype [45].

The taxonomic scope of the present paper is far broader. We examined morphometrically a much larger number of plants (457) that collectively span 113 of the 353 microspecies and all 29 of the mesospecies recognised by Delforge [39]; these plants adequately represent the variation found within all nine of the molecularly circumscribed macrospecies recognised by Bateman et al. [43]. Our sampling strategy was designed to explore every level in the demographic hierarchy, downwards from macrospecies > mesospecies > microspecies > population > individual plant within population, in order to test Bateman’s [28,29,45] continuum hypothesis of variation within macrospecies. Given that species are the most fundamental units in our attempts to understand evolution and ecology, to conserve nature and to predict the consequences of future environmental changes, it is our contention that species should be self-circumscribing “natural” entities rather than mere conveniences of classification.

Taking as read the nine essentially self-circumscribing molecular macrospecies of *Ophrys* (Figure 1), we sought evidence that might suggest the existence of self-circumscribing entities within those macrospecies—in other words, taxa that could allow a more finely resolved natural classification based on some kind of mesospecies concept. We also sought repeated patterns of correlation among the 51 characters scored—patterns that could credibly be interpreted as adaptive trends resulting from directional or disruptive selection, critically reappraising evidence that *Ophrys* is presently in the midst of an evolutionary radiation. We used this case-study to review more broadly the strengths and weaknesses of our preferred morphometric approach, which we have applied to seven Eurasian orchid genera since it was devised by Bateman and Denholm [56] more than 40 years ago, before returning to a popular recent debate—the most appropriate role to award evolutionary mechanisms when attempting to optimise taxonomy.

Throughout the text, epithets representing the macrospecies of Bateman et al. [43] are presented in roman script with a capitalised first letter. In contrast, the mesospecies and microspecies of Delforge [39] are italicised; mesospecies epithets have a capitalised first letter, whereas microspecies epithets are presented entirely in lower case (they are usually also preceded by ‘*O*.’).

## 2. Materials and Methods

### 2.1. Plant Materials

Fieldwork for our broader research programme targeted specifically at the genus *Ophrys* began in 2004 in the Peloponnese and ended in 2019 on Rhodes; populations were sampled across most of the geographic range of the genus, excepting only the extreme East in the Levant and the Caucasus. Initially, morphometric analyses were confined to macrospecies Fuciflora and Sphegodes, but we later expanded our taxonomic coverage; the last macrospecies to be included in our study was Fusca, sampled from 2008 onward.

Our standard procedure has for long been to randomly select study plants within field populations and to measure their vegetative characters in situ, also obtaining 1:1-scale perpendicular and lateral images of a representative flower taken from each plant as a record of its morphology and potential source of coordinates for use in geometric morphometrics [66,67,68]. Sampling is thus confined to excising one flower for mounting and morphometric study later in the same day while placing a second flower in a sachet of fine-ground silica gel to permit subsequent DNA analyses. However, our aims in most previous morphometric studies of European orchids have been to typically sample ten plants per population and ten or more populations per putative species [66,69]. In this study, several factors, most notably the vast number of microspecies described within the genus *Ophrys*, necessitated cruder, more pragmatic sampling that tolerated not only much smaller sample sizes per putative microspecies but also greater heterogeneity of sampling among microspecies.

### 2.2. Morphometric Data Collection

**Figure 2:**
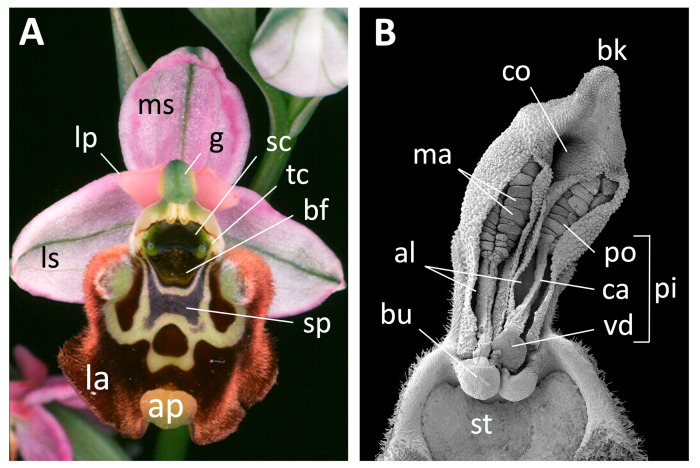
Terminology of a bee orchid flower. (**A**) Floral features, exemplified by microspecies *O. episcopalis* (mesospecies *Fuciflora*, macrospecies Fuciflora). (**B**) Gynostemium features, exemplified by *O. cretensis* (mesospecies *Mammosa*, macrospecies Sphegodes). Labels on (**A**): la, labellum (lip); lp, lateral petal; ms, median sepal; ls, lateral sepal; g, gynostemium (column); sc, stigmatic cavity; tc, temporal callosity (“pseudoeye”); bf, basal field; sp, speculum; ap, appendix. Labels on (**B**): al, anther locules; bu, bursicle (enclosing viscidial disc); bk, beak; co, connective; st, stigmatic surface; po, pollinium; ma, massulae; ca, caudicle; v, viscidial disc (these features are collectively termed the pollinarium–pi). Images: A = Richard Bateman, B = Paula Rudall.

**Figure 3:**
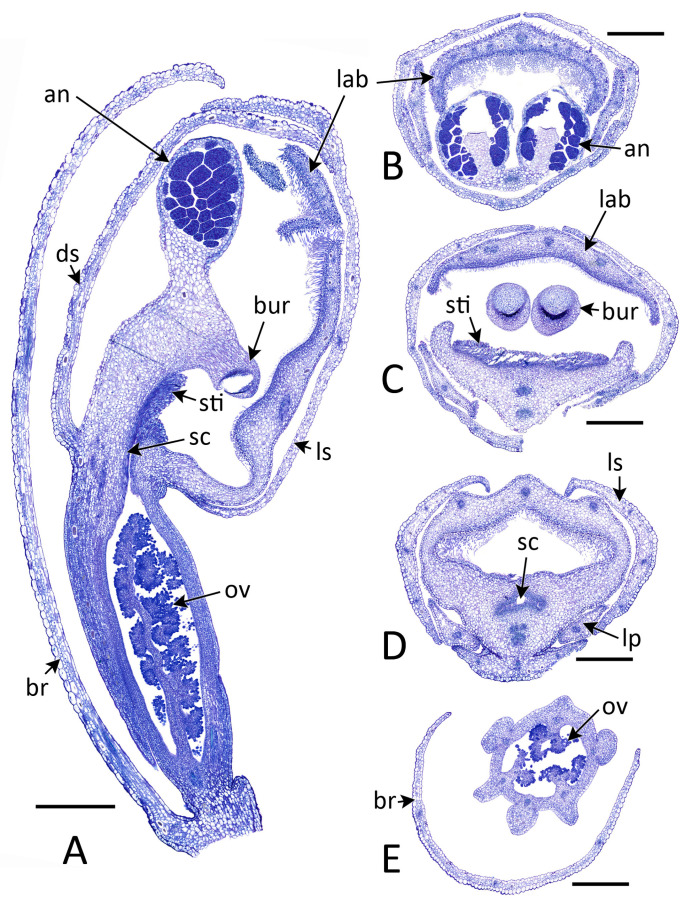
Anatomical sections of pre-anthetic flower bud of *O. pallida* (mesospecies *Obaesa*, macrospecies Fusca). (**A**) Median longitudinal section, showing entire bud subtended by bract. The anther, stigma and prominent bursicles are enclosed on one side by the labellum (which become strongly recurved once the flower has opened); it bears localised projecting trichomes. Note the exceptionally narrow stylar canal below the stigmatic papillae—a constriction encouraging pollen-tube competition. The elongate ovary contains numerous tiny ovules borne on branched placentae. Nectaries are absent. (**B**–**E**) Transverse sections. (**B**) Distal region, showing the anther, which contains dark-staining massulae, and the labellum, which bears outward- and backward-projecting trichomes. (**C**) Central region, showing the stigma and the lower parts of the prominent bursicles. (**D**) Proximal region, showing the narrow stylar canal. (**E**) Unilocular ovary containing many tiny ovules borne on branched parietal placentae. Labels: an, anther; bur, bursicle; br, bract; lab, labellum; lp: lateral petal; ls, lateral sepal; ov, ovules; sc, stylar canal; sti, stigma. All scales = 1 mm. All images: Paula Rudall.

Morphometric characters employed in the present study are listed in Appendix A (for terminology see also Figure 2 and Figure 3). Our initial list of 53 characters included two microscopic characters describing marginal bract cells, but these characters quickly proved to be insufficiently informative relative to the considerable amount of time consumed in recording them; hence, they were soon discarded. The remaining 51 characters contributing to the statistical analyses describe the stem and inflorescence (5), leaves and bracts (7), gynostemium and ovary (3), labellum (20) and lateral petals and sepals (16). They can alternatively be categorised as metric (33), meristic (3), multistate-scalar (13) and bistate (2). Flower colour was recorded by matching the colour of the lower half of the labellum (excluding the speculum), the sepals and the lateral petals to the closest colour block(s) of the Royal Horticultural Society Colour Chart for subsequent conversion into three quantified variables long recognised by the Commission Internationale de l’Eclairage.

Data for individual plants were summarised in an Excel v15.4 spreadsheet. Two rounds of multivariate data analysis were performed. The first, stand-alone analysis involved the complete matrix of 457 individuals (summarised in Table 1), together encompassing all 29 of the mesospecies and 113 (32%) of the 353 microspecies listed by Delforge [39].

The complete morphometric matrix of 457 plants × 51 characters (total 23,307 cells) contained 4.4% (1026) missing values—a figure that fell to just 0.7% (120) if only the 39 floral characters were considered. The most frequent cause of missing values among vegetative characters was the premature desiccation of the leaves, a phenomenon that affects plants growing in an unusually arid environment or sampled during an unusually arid spring (most commonly affecting macrospecies Sphegodes and Apifera).

### 2.3. Morphometric Data Analysis

For all matrices and submatrices, the assembled data were analysed via multivariate methods using Genstat v14 [70]. They were employed to compute a symmetrical matrix that quantified the similarities of pairs of datasets (i.e., plants) using the Gower Similarity Coefficient [71] on unweighted datasets scaled to unit variance. The matrix was in turn used to construct a dendrogram and a minimum spanning tree [72] and subsequently to calculate principal coordinates [73,74]—compound vectors that incorporate positively or negatively correlated characters that are most variable and therefore potentially diagnostic of putative taxa. As discussed in greater detail in Section 4.1, principal coordinates are especially effective for simultaneously analysing heterogeneous suites of morphological characters and can comfortably accommodate missing values. They have proven invaluable for assessing relationships among orchid species and populations throughout the last three decades (reviewed by Bateman [29,67,68]) and are the crux of the morphometric element of the present study.

For each multivariate analysis, the first four principal coordinates (PC1–4) were plotted together in pairwise combinations to assess the degree of the morphological separation of individuals (and thereby of populations and taxa) in these dimensions, and pseudo-F statistics were obtained to indicate the relative contributions to each coordinate of each of the original variables. The resulting ordinations were presented using Deltagraph v7.1 (SPSS/Red Rock software). In our many previous morphometric studies of orchids, we have also presented data based on overall similarity values (i.e., dendrograms and/or minimum spanning trees), but herein, we have focused exclusively on ordinations (a) because they have reliably proved to be more taxonomically discriminatory, (b) because we wished to compare ordinations resulting from alternative combinations of characters and ratios, and (c) because we wished to label some of the ordinations according to three contrasting categories: geographic origin, mesospecies assignment and microspecies identity.

Each of the 447 plants measured was attributed to one of the nine molecularly circumscribed macrospecies. Wherever possible, individuals were assigned to macrospecies on the basis of DNA data presented in previous studies [19,20,21,24,43,44,75], but where this was not possible, they were assigned on the basis of the closest morphological match that had benefited from molecular analysis. We recognise that this system is not wholly infallible, as was demonstrated by the supposed Greek local endemic microspecies *O. delphinensis*; when analysed using RAD-seq, this microspecies proved to be of recent hybrid origin, having a phenotype closer to that of macrospecies Sphegodes but a maternally inherited plastid genotype typical of macrospecies Fuciflora [45].

Each of the nine macrospecies was analysed separately. For the four macrospecies that have not (yet) been split taxonomically into numerous microspecies (Insectifera, Speculum, Bombyliflora, Apifera) and hence are represented herein by only small numbers of individuals, only two analyses were conducted: the full suite of 51 characters and a more restricted character-set consisting only of the 39 floral characters. The motivations for experimenting with removing the 12 vegetative characters (i.e., characters C40–51) were the facts that (a) these characters incurred higher frequencies of missing values, and (b) in terrestrial orchids, vegetative organs consistently show considerably greater epigenetic and ecophenotypic variation [76]. For each of the five macrospecies that have been split into numerous microspecies—and in most cases into several mesospecies (i.e., macrospecies Tenthredinifera, Fusca, Umbilicata, Fuciflora, Sphegodes)—and hence are represented herein by between 31 and 137 individuals (Table 1), further analyses were conducted wherein all metric floral measurements had been converted to ratios, a transformation enacted in order to minimise the impact of overall size differences on the resulting ordinations. All ordinations were labelled according to both microspecies identity and geographic origin, but only those groups rich in microspecies required additional categorisation according to mesospecies assignment.

Finally, similar analytical approaches were applied to a master matrix combining data for all nine macrospecies (also a submatrix consisting of the three most closely related macrospecies), with the aim of identifying the strongest patterns of morphological variation evident across the entire genus *Ophrys*. This ordination was labelled according to macrospecies, mesospecies and geographic origin. In addition, a second genus-wide ordination was conducted after reducing all metric measurements in the matrix to ratios, aiming to maximise contrasts in the shape of various organs while minimising the influence of plant size and vigour.

### 2.4. Scanning Electron Microscopy and Anatomy

Flowers of 12 selected microspecies collectively representing seven of the nine macrospecies were either collected by us in the field (most in Crete in 2017) or, in five cases, selected from among samples previously collected by knowledgeable individuals and deposited in the Spirit Collection of the Royal Botanic Gardens Kew.

Preparation for scanning electron microscopy involved selecting flowers from each inflorescence for dehydration through an alcohol series to 100% ethanol. They were then stabilised using an Autosamdri 815B critical-point drier, mounted onto stubs using double-sided adhesive tape, coated with platinum using an Emtech K550X sputter-coater, and examined under a Hitachi cold-field emission SEM S-4700-II at 2 kV. The resulting images were recorded digitally for subsequent aggregation in Adobe Photoshop.

In addition, flowers of a representative member of macrospecies Fusca from Sicily were prepared for anatomical light microscopy. Alcohol-fixed flowers were transferred through an ethanol series to 100% ethanol, followed by an ethanol–LR-White resin series, then embedded in LR-White resin using a vacuum oven set at 60 °C. Semi-thin sections were cut using a Reichert-Jung Ultracut ultramicrotome and a glass knife before mounting on glass slides. Sections were stained with Alcian Blue and imaged using a Leica DM6000B light microscope.

## 3. Results

### 3.1. Scanning Electron Microscopy and Anatomy

Two morphological extremes within the genus *Ophrys* are illustrated in Figure 2A and Figure 3, together showing all of the relevant features of bee orchids. Both flowers show the classic orchid architecture of two closely spaced, alternating whorls of sepals and petals, the median petal forming a larger and more elaborate labellum that, along with the fused gynostemium into which it grades, is responsible for much of the functional morphology of the flower. The labellum is spur-less and dark brown; it varies in outline and three-dimensional topography, in the size and distribution of trichomes, and in the size and complexity of the paler trichome-free region termed a speculum. A distal appendix and lateral ‘horns’ may be present (Figure 2A) or absent (Figure 3). The lateral petals are considerably shorter and narrower than the sepals; all five tepals are typically coloured green or, less often, pink–purple.

**Figure 4:**
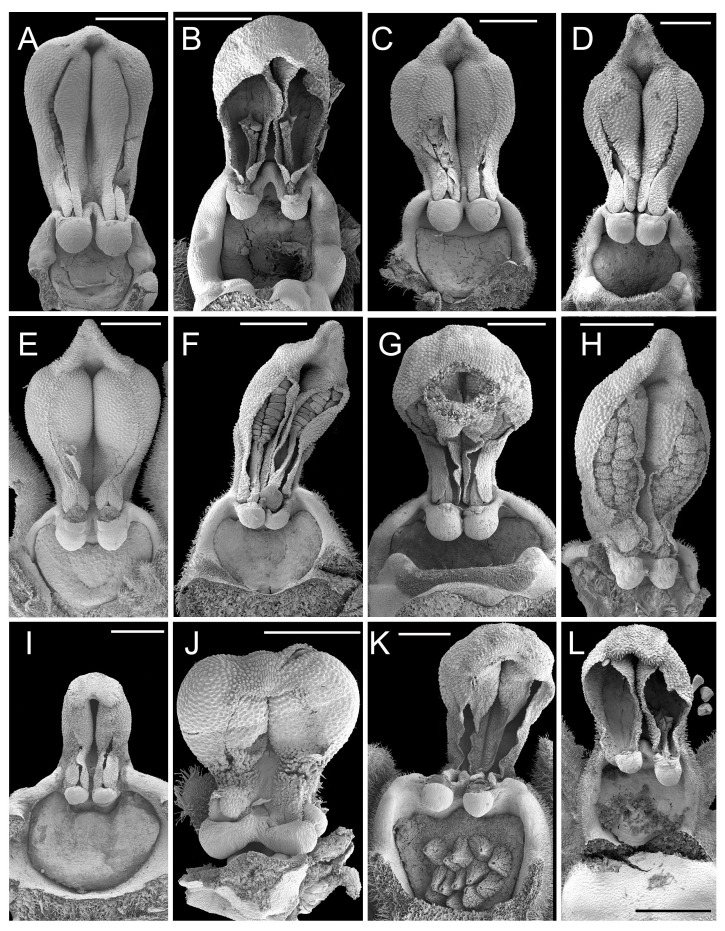
Scanning electron micrographs of gynostemia of 12 *Ophrys* microspecies, collectively representing seven of the nine macrospecies. (**A**) *O. vernixia*: mesospecies *Speculum*, macrospecies Speculum. (**B**) *O. regis-ferdinandii*: mesospecies *Speculum*, macrospecies Speculum. (**C**) *O. ferrum-equinum*: mesospecies *Mammosa*, macrospecies Sphegodes. (**D**) *O. heldreichii*: mesospecies *Heldreichii*, macrospecies Fuciflora. (**E**) *O. mammosa*: mesospecies *Mammosa*, macrospecies Sphegodes. (**F**) *O. cretensis*: mesospecies *Mammosa*, macrospecies Sphegodes. (**G**) *O. lacaena*: mesospecies *‘Bornmuelleri’*, macrospecies Fuciflora. (**H**) *O. sphegodes*: mesospecies *Sphegodes*, macrospecies Sphegodes. (**I**) *O. phryganae*: mesospecies *Lutea*, macrospecies Fusca. (**J**) *O. bombyliflora*: mesospecies *Bombyliflora*, macrospecies Bombyliflora. (**K**) *O.* cf. *leochroma*: mesospecies *Tenthredinifera*, macrospecies Tenthredinifera. (**L**) *O. insectifera*: mesospecies *Insectifera*, Macrospecies Insectifera. Scale in all images = 1 mm. Images: Paula Rudall.

Figure 3 and Figure 4 show that, in most of its characteristics, the gynostemium of *Ophrys* is typical of other closely related genera within the subtribe Orchidinae. The paired, elongate anther-sacs are near-parallel and closely juxtaposed, their length typically exceeding considerably the diameter of the underlying stigmatic surface. Each loculus contains a single tripartite, club-shaped pollinarium. The distal pollinium typically bears 50–100 massulae, each consisting of many tightly packed pollen tetrads; it is linked to the proximal adhesive viscidial disc by a caudicle of similar length (Figure 4B). The viscidium is enclosed in a protective, desiccation-resistant bursicle. More distinctive of the genus *Ophrys* is the fact that, although closely juxtaposed and rather scrotal in appearance, the bursicles are actually separate, allowing (and perhaps even encouraging) a visiting insect to remove only one of the two pollinaria. The stigmatic surface is more concave, equidimensional and simpler in outline than those of many other Orchidinae. The narrow stylar canal links the stigmatic surface to the unilocular ovary (Figure 3A,D), guiding innumerable pollen tubes towards similarly large numbers of minute ovules. *Ophrys* massulae are unusually cohesive, as demonstrated by the remains of a disaggregated pollinium that are attached to the stigmatic surface of the *O. leochroma* flower shown in Figure 4K.

However, there are relatively few differences among the viscidia of contrasting macrospecies within the genus *Ophrys* (Figure 4). It has long been known [43] that a clade consisting of five macrospecies (groups F–J in Figure 1) is characterised by the possession of a triangular beak extending upwards from the connective (Figure 4C–H, though almost severed in G). This extension reaches its maximum expression in the “flying duck” gynostemium of macrospecies Apifera (not shown). However, Figure 4 usefully increases the number of autapomorphic states recognised in macrospecies Insectifera, specifically by revealing the presence of a thickened, strongly papillose connective that resembles a toupé (Figure 4L). The anthers appear comparatively short in the macrospecies Insectifera (Figure 4L), Fusca (Figure 4I) and especially Bombyliflora (Figure 4J) but more elongated in the macrospecies Speculum (Figure 4A,B). The stigmatic surface is unusual in being slightly higher than wide in two macrospecies, Insectifera (Figure 4L) and Speculum (Figure 4A,B), and is especially deeply recessed—and hence particularly well-defined—in the Fusca group (Figure 4I).

### 3.2. Morphometrics: Analyses Involving Multiple Macrospecies

#### 3.2.1. All Nine Macrospecies

**Figure 5:**
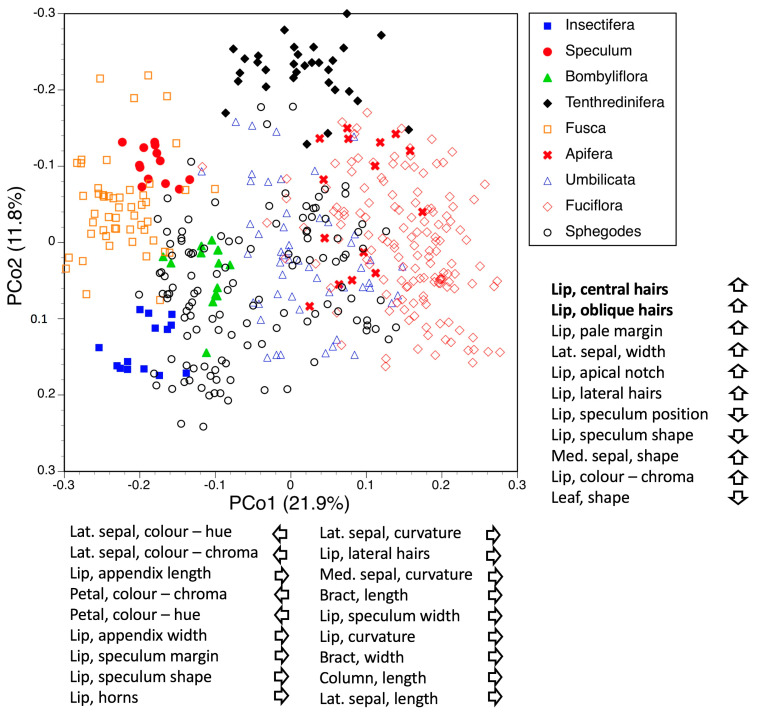
Plot of principal coordinates 1 and 2 for the genus *Ophrys*, labelled according to nine macrospecies groups: 447 individuals from ca. 300 localities, 51 variable characters. Characters contributing to each coordinate are listed in descending order, with arrows indicating the direction of increase in the value of each character (boldface characters dominate that coordinate).

Seeking a preliminary overview, our initial analysis used all 457 individuals and all 51 characters (Figure 5). The first two principal coordinates encompassed 34% of the total variation, the first coordinate being considerably stronger than the second. As expected, given the extreme multi-directionality of character variation within the genus, the analysis failed to yield the complete separation of any of the nine macrospecies from the remainder, though it did helpfully suggest polarisation between two morphological extremes. The first coordinate gave almost complete separation of a group of four comparatively cohesive macrospecies (Insectifera, Fusca, Speculum, Bombyliflora) that reliably possess green (rather than pink) lateral petals and sepals and a labellum that shows little or no expression of an appendix, bears a relatively simple speculum and (with the exception of Bombyliflora) tends to be comparatively two-dimensional. Fuciflora and Apifera constitute the opposing pole of PCo1, leaving in intermediate positions Tenthredinifera, Umbilicata and Sphegodes; the latter macrospecies appears especially incohesive.

The second coordinate yielded the almost complete separation of Tenthredinifera from the remainder, based mainly on its trichome-rich, apically notched labellum, featuring a yellow marginal zone and a small and simple but prominent speculum. These characters also allow some differentiation among macrospecies Insectifera, Bombyliflora, Speculum and Fusca, though plants of the latter are more widely spread due to their considerable variation in flower size and trichome development.

Surprisingly, the third coordinate offered no taxonomic separation—unfortunately, it was dominated by a character (C49) that was conceived with the intention of describing leaf shape but proved to be overly crude. The fourth coordinate (not shown) used characters such as petal curvature, speculum length and the presence versus absence of brown stripes on the lateral sepals to establish polarity between Speculum at one extreme and Insectifera at the other, but otherwise this axis was not taxonomically discriminatory.

#### 3.2.2. The Umbilicata-Fuciflora-Sphegodes Clade

**Figure 6:**
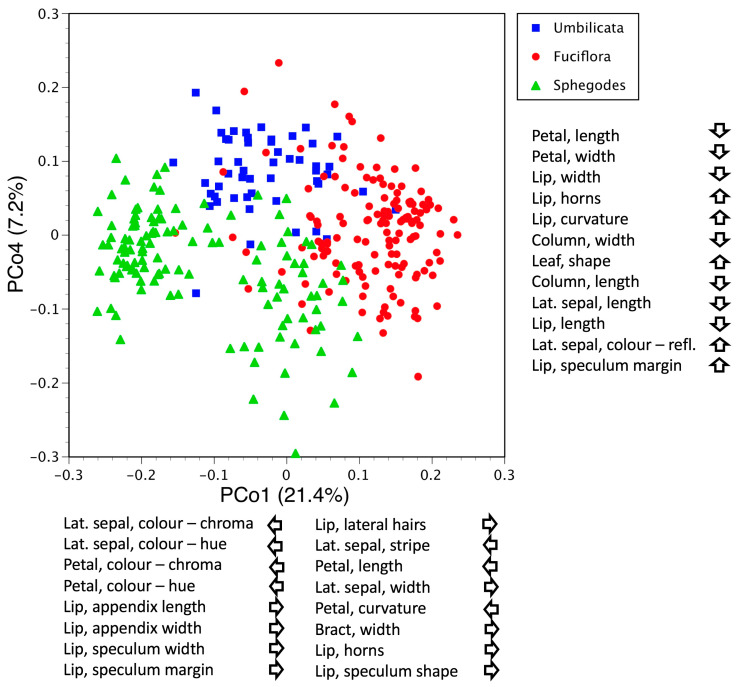
Plot of principal coordinates 1 and 4 for the macrospecies Umbilicata plus Fuciflora plus Sphegodes, labelled according to three macrospecies groups: 319 individuals from ca. 200 localities and 51 variable characters. Characters contributing to each coordinate are listed in descending order, with arrows indicating the direction of increase in the value of each character.

In the hope of reducing dimensionality in the data, we therefore attempted an analysis of three of the four macrospecies most intensively sampled here (Figure 6), which (as determined in our RAD-seq tree; Figure 1) together form a discrete, relatively tight-knit clade: Umbilicata, Fuciflora and Sphegodes. Based on analysis of 319 plants, the first coordinate largely separated most Fuciflora from the majority of Sphegodes plants, with Umbilicata intermediate. However, Sphegodes plants were split into two groups of approximately equal numbers of plants, based largely on whether their petals and sepals were green or pink. Similarly, green-flowered Fuciflora plants (a minority within this macrospecies) were consequently placed closer to Sphegodes.

We anticipated that re-analysing this matrix after removing the six characters that represent petal and sepal colour would unify the two groups of Sphegodes, which it did, and would thereby reduce the overall morphological overlap of Sphegodes with Fuciflora, which it did not (results not shown). In fact, the amount of overlap increased. Evidently, flower colour is in fact important for distinguishing the macrospecies, but it largely operates at a level that is more subtle than simply contrasting green with pink. Clearly, the presence of colour dimorphism within each of these macrospecies is problematic with regard to taxon circumscription at finer scales. Nonetheless, additional characters contributing to the first coordinate, such as the presence and size of a labellar appendix, nature of the speculum, petal length and sepal width, also contribute considerably to distinguishing the three macrospecies in this analysis.

The second and third coordinates were much weaker than the first and provided little taxonomic separation. Only when reaching down to the fourth coordinate (Figure 6) was Umbilicata partially separated from Fuciflora and Sphegodes, on the basis of its smaller petals and columns, and (for many Umbilicata microspecies) also the narrower, more three-dimensional labella bearing prominent horns.

### 3.3. Morphometrics: Analyses Involving Single Macrospecies but Multiple Mesospecies

#### 3.3.1. Macrospecies Sphegodes

**Figure 7:**
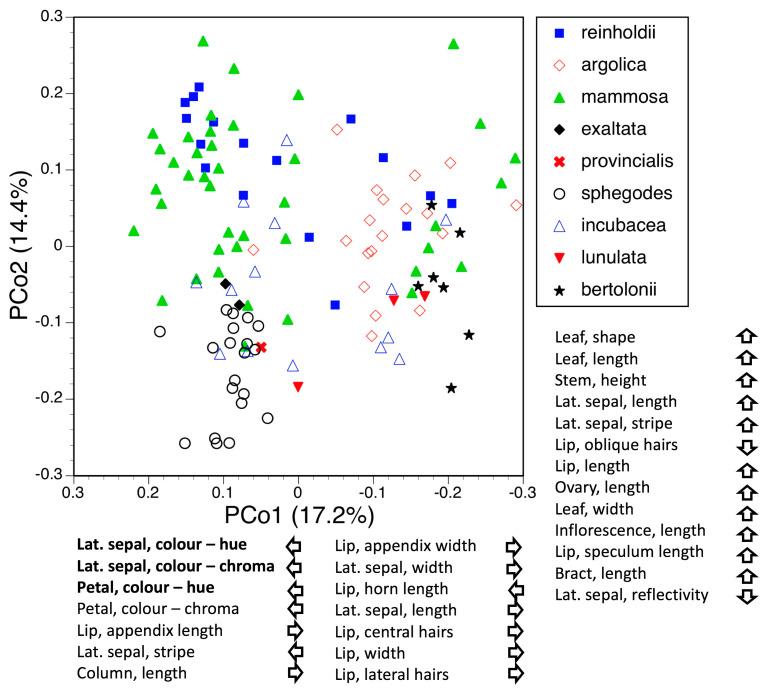
Plot of principal coordinates 1 and 2 for the macrospecies Sphegodes, labelled according to nine mesospecies groups: 124 individuals from 104 localities and 51 variable characters. Characters contributing to each coordinate are listed in descending order, with arrows indicating the direction of increase in the value of each character (boldface characters dominate that coordinate).

The first two principal coordinates for the morphometric matrix of 124 plants of macrospecies Sphegodes are plotted together in Figure 7, individual plants being labelled according to mesospecies assignment sensu Delforge [39]. Although totalling only 32% of the total variance, the first two coordinates are considerably stronger than the remainder. The first coordinate is dominated by the contrast between plants with green (left) versus pink (right) lateral petals, which again divides the plants into two ill-defined clusters. Subordinate contributory characters show that plants located toward the right of the plot typically possess labellar appendixes and have comparatively long columns, large sepals and labella that are comparatively hairy but tend to lack forward-pointing horns. The more egalitarian second coordinate represents several characters that contribute approximately equally, together reflecting comparatively large plant size and, to a lesser degree, large flower size—effectively constituting a vigour coordinate. The third coordinate combines other vegetative dimensions and leaf number with a mixed bag of labellum characters. All coordinates of fourth order and below reflect very few characters and offer little if any taxonomic discrimination.

Viewed at the level of mesospecies, the impact of the first coordinate (Figure 7) is overly dependent on whether the mesospecies in question encompasses a mixture of green- and pink-petaled plants; consequently, those mesospecies considered capable of exhibiting both colours (*Mammosa*, *Incubacea*, *Reinholdii*) are spread more widely along the first coordinate than are those that are either reliably green (all lack anthocyanins, e.g., *Sphegodes*) or reliably pink (all possess anthocyanins, e.g., *Bertolonii*). The second coordinate gives the almost complete separation of the large-bodied, large-flowered mesospecies *Mammosa* and *Reinholdii* from the more modestly sized mesospecies *Sphegodes* and *Provincialis*. The third coordinate serves only to partially separate mesospecies *Incubacea* from the remainder (not shown).

In an additional experiment, morphological variation within populations of a single microspecies was estimated through analysis of 15 pairs of con-microspecific plants that together encompassed the full morphological range exhibited by macrospecies Sphegodes. Distances separating these paired individuals on Figure 7 varied greatly from less than 0.01 to 0.21, averaging 0.050 ± 0.046 for PCo1 and 0.079 ± 0.061 for PCo2. The comparatively large mean value for this comparison on the second coordinate relative to the first coordinate is readily explained by contrasts in plant size encapsulated by PCo2 that are likely to reflect differences in the development (ontogeny) and environment of growth (ecophenotypy) at least as much as any direct genetic influence (see also [45]).

#### 3.3.2. Macrospecies Fuciflora

**Figure 8:**
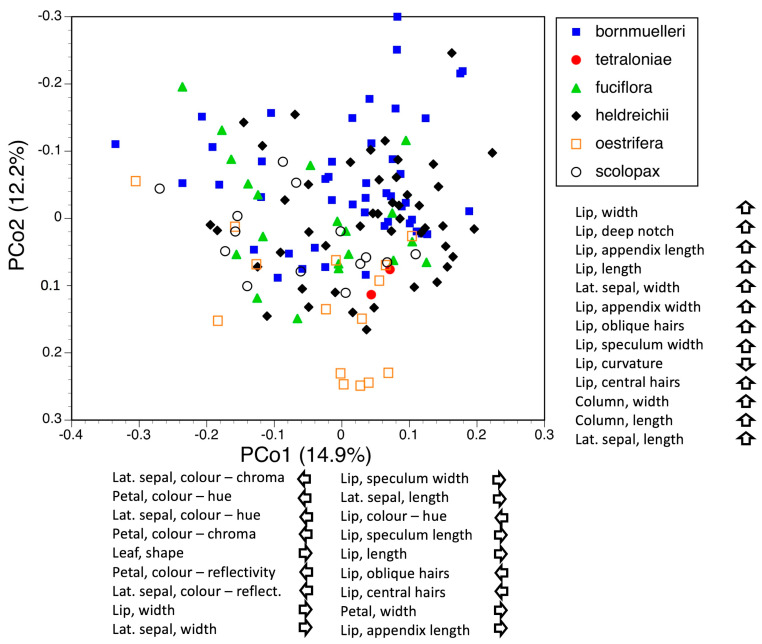
Plot of principal coordinates 1 and 2 for the macrospecies Fuciflora, labelled according to six mesospecies groups: 143 individuals from 68 localities and 51 variable characters. Characters contributing to each coordinate are listed in descending order, with arrows indicating the direction of increase in the value of each character (see also Figure 16).

The first two coordinates for the 143 plants of Fuciflora represent just 27% of the total variance (Figure 8), and neither is effective at distinguishing among the six sampled mesospecies. As with Sphegodes, the first coordinate is dominated by sepal and petal colour, supported by various flower size characters plus leaf shape. Most of the characters contributing to the second coordinate are also positively correlated dimensions of various floral parts, suggesting that much of the morphological variation within macrospecies Fuciflora reflects differences in flower size.

The third and fourth coordinates proved to be more discriminatory at the mesospecies level. The third coordinate almost completely separated *Fuciflora* and *Tetraloniae* from most of the remaining mesospecies on the basis of their poorly developed labellar horns and shoulder hair, larger petals and modest vegetative size. The fourth coordinate partially separated mesospecies *Heldreichii* and *Scolopax* from the remainder on the basis of their laterally reflexed labella with prominent lateral lobes and their comparatively low vegetative vigour.

A finer-scale analysis of this relatively well-sampled macrospecies is presented in Section 3.5.

#### 3.3.3. Macrospecies Umbilicata

**Figure 9:**
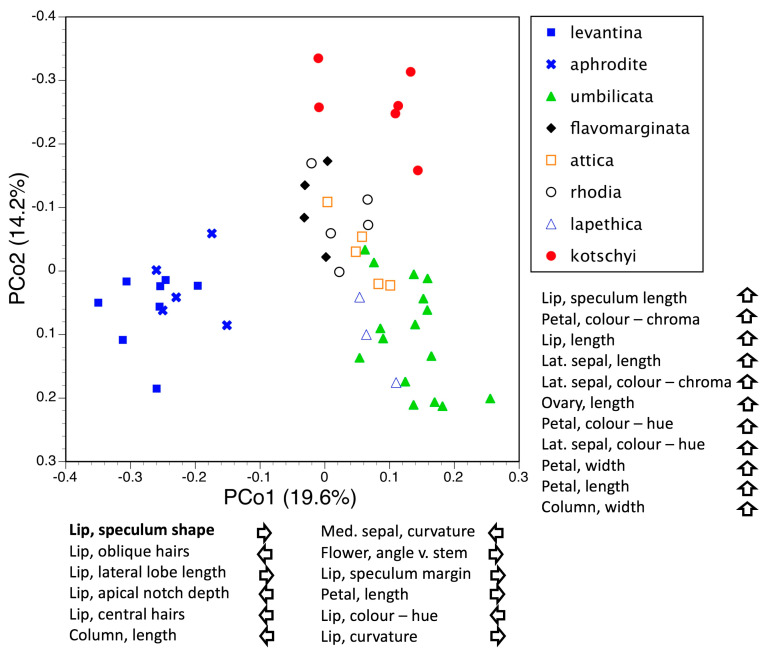
Plot of principal coordinates 1 and 4 for the macrospecies Umbilicata, labelled according to eight microspecies groups: 52 individuals from 44 localities and 51 variable characters. Characters contributing to each coordinate are listed in descending order, with arrows indicating the direction of increase in the value of each character (boldface characters dominate that coordinate).

The eastern Mediterranean macrospecies Umbilicata was analysed for 52 plants representing two mesospecies and eight microspecies. This plot differed from almost all of the other analyses performed in that the plants were resolved by the first coordinate into two discrete groups separated by a genuine discontinuity (Figure 9). Specifically, the Cypriot microspecies *levantina* and *aphrodite* were distinguished from the remainder primarily as a result of their simpler, less striking specula, supported by characters demonstrating that their labella were also more hirsute and less three-dimensional, with little if any development of lateral lobes, and were more likely to develop apical notches; they were also more likely to be presented parallel to the stem.

The second coordinate organised the remaining six microspecies into a near-linear arrangement of three crude clusters: *umbilicata* plus *lapethica*, *attica* plus *flavomarginata* plus *rhodia*, and *kotschyi*. This cline represents two correlated trends: from strong pink through pink-washed green to strong green sepals and petals, and from smaller to larger flowers, especially with regard to labellum length.

The third axis almost completely separated microspecies *aphrodite*, *attica* and *rhodia* from the remainder on the basis of their wider sepals and often a lack of a brownish–pink wash on the petals, whereas in contrast the fourth axis reflected vegetative vigour, thereby separating *aphrodite* from the more modestly proportioned *levantina* (not shown).

#### 3.3.4. Macrospecies Tenthredinifera

**Figure 10:**
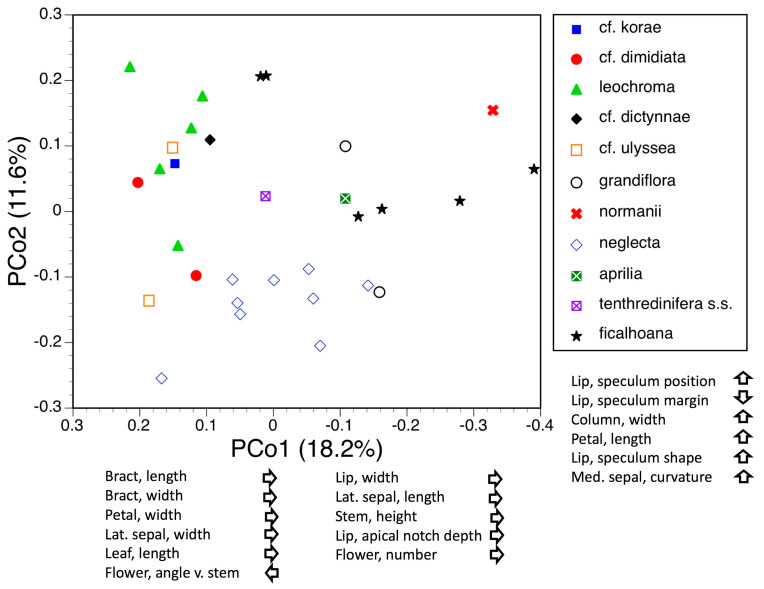
Plot of principal coordinates 1 and 2 for the macrospecies Tenthredinifera, labelled according to nine mesospecies groups: 31 individuals from 27 localities and 47 variable characters. Characters contributing to each coordinate are listed in descending order, with arrows indicating the direction of increase in the value of each character.

Macrospecies Tenthredinifera is represented by 31 plants encompassing 11 (mostly recently conceived) microspecies. The first coordinate largely separated *normanii*, *grandiflora* and *ficalhoana* from the remaining microspecies (Figure 10). It reflected their combination of vegetative vigour, notably larger bracts and leaves, and floral characters such as lateral sepal size, labellum width and the presentation of the flowers parallel to the stem. The considerably weaker second coordinate provided little discrimination, other than partially separating the Sardinio–Corsican microspecies *neglecta* according to its simple, basally concentrated, brightly margined speculum, short petals and comparatively narrow column.

The third coordinate, mainly reflecting the reflectivity and hue of the lateral sepals, offered no taxonomic discrimination, whereas the fourth coordinate largely separated the Sardinian microspecies *grandiflora* and *normanii* from the remaining nine microspecies analysed, primarily because they possessed ovate rather than obovate median sepals.

#### 3.3.5. Macrospecies Fusca

**Figure 11:**
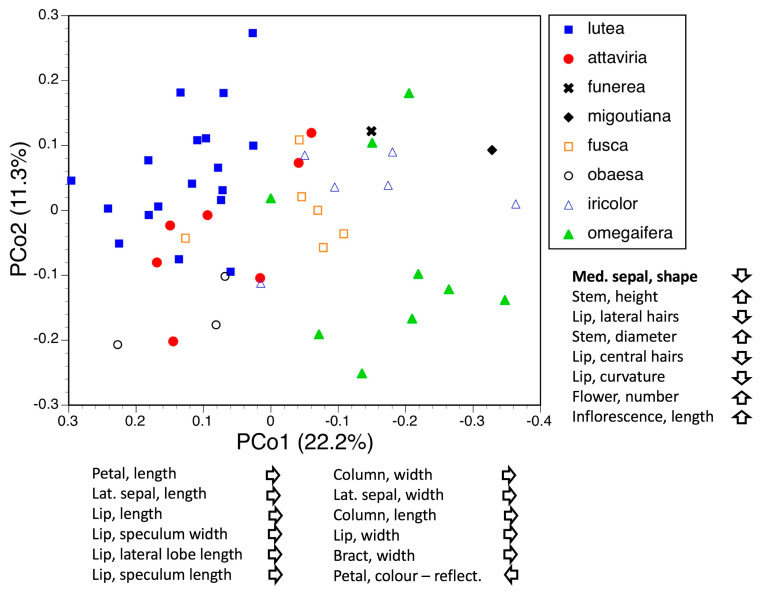
Plot of principal coordinates 1 and 2 for the macrospecies Fusca, labelled according to eight mesospecies groups: 51 individuals from 46 localities and 46 variable characters. Characters contributing to each coordinate are listed in descending order, with arrows indicating the direction of increase in the value of each character (boldface characters dominate that coordinate).

The 51 plants (and 33 microspecies) of macrospecies Fusca yielded a first coordinate that represented correlated size contrasts in all floral organs (Figure 11), mesospecies *Lutea*, *Attaviria* and *Obaesa* having the smaller flowers. The second coordinate represented a negative correlation between increases in several plant size characters versus increased hairiness of labellum and possession of an obovate median sepal; short, few-flowered plants with relatively trichome-rich labella characterise mesospecies *Omegaifera* and *Obaesa*. Neither the third coordinate, dominated by leaf shape, nor the fourth coordinate, dominated by the width, colour (chroma) and pale margin of the labellum, provided meaningful distinction among mesospecies.

Because the first two coordinates relied so heavily on labellar dimensions, we re-analysed the matrix after replacing 18 metric measurements with 10 ratios derived from those metric measurements (not shown), aiming to emphasise the shapes of structures rather than their relative sizes. This seemingly radical modification imposed on the underlying data had surprisingly little impact on the revised distribution of plants across the first two coordinates; moreover, it neither strengthened nor weakened the clustering of individuals according to mesospecies assignment.

### 3.4. Morphometrics: Analyses Involving Single Macrospecies and Single Mesospecies

#### 3.4.1. Macrospecies Speculum

**Figure 12:**
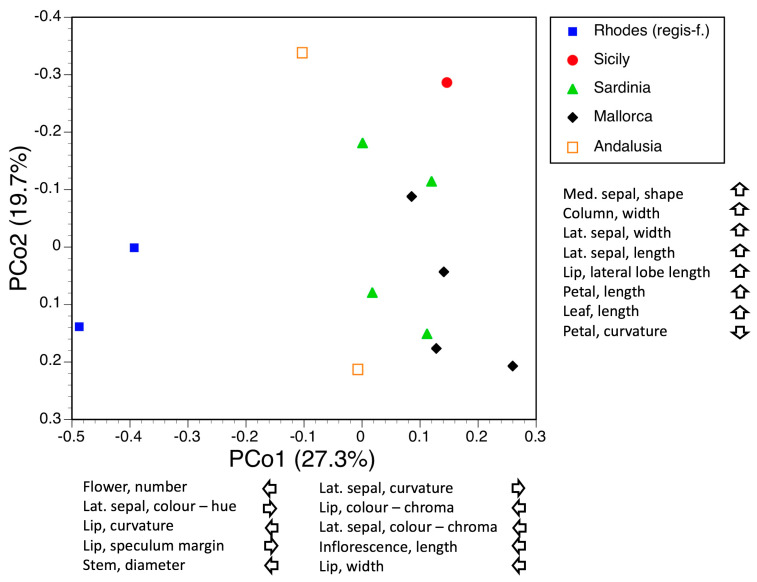
Plot of principal coordinates 1 and 2 for the macrospecies Speculum, labelled according to five regions of origin: 13 individuals from 12 localities and 42 variable characters. Characters contributing to each coordinate are listed in descending order, with arrows indicating the direction of increase in the value of each character.

Although dominated by *O. speculum* s.s., this small-scale analysis of 13 plants from 12 localities spanning the Mediterranean also included two plants of the Eastern microspecies *O. regis-ferdinandii* (Figure 12). The two microspecies were readily separated on the first coordinate, *regis-ferdinandii* tending to produce a larger number of flowers that possessed a lip with strongly recurved lateral margins and lateral sepals that curved more strongly forwards and bore broader brown longitudinal stripes. All lower-order coordinates failed to distinguish among plants of *O. speculum* s.s. from contrasting regions of the Mediterranean Basin, the two plants sampled in Andalusian Spain being especially widely separated from each other on the second coordinate. This result discourages the division of *O. speculum* into further microspecies.

#### 3.4.2. Macrospecies Bombyliflora

**Figure 13:**
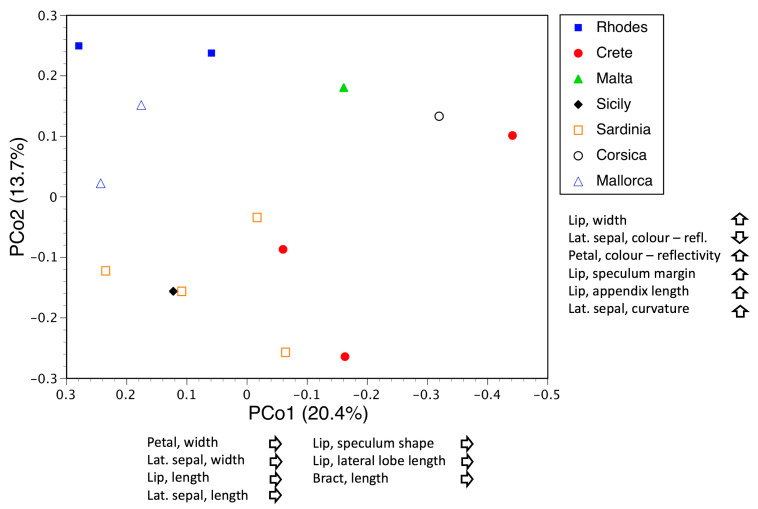
Plot of principal coordinates 1 and 2 for the macrospecies Bombyliflora, labelled according to four regions of origin: 14 individuals from 14 localities and 47 variable characters. Characters contributing to each coordinate are listed in descending order, with arrows indicating the direction of increase in the value of each character.

Thus far, macrospecies Bombyliflora has escaped the taxonomic fragmentation into microspecies that has afflicted most other macrospecies of *Ophrys.* Nonetheless, we were interested to see whether any correlated trends might emerge from our small but geographically extensive set of plants: 14 plants from 14 localities, sampled on seven islands that together span the entire Mediterranean. The four islands that yielded multiple datasets suggest that, as expected, morphological variation on particular islands is less than that encompassed by the macrospecies as a whole. Nonetheless, no geographical clines are evident, epitomised by the fact that plants from the easternmost and westernmost of the islands sampled are placed together in the top-left region of the ordination (Figure 13). Again, division into microspecies appears unjustified.

The first coordinate largely reflects flower size. The second coordinate is less thematic, but examination of the underlying data showed that the heterogeneous suite of floral characters vary independently rather than in concert. The third and fourth coordinates are uninformative, being dictated by single characters: leaf shape and flower number, respectively.

#### 3.4.3. Macrospecies Apifera

**Figure 14:**
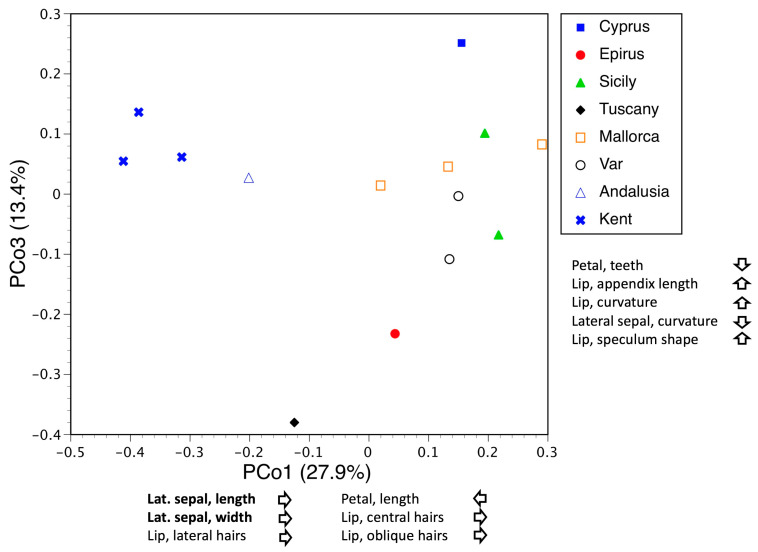
Plot of principal coordinates 1 and 3 for the macrospecies Apifera, labelled according to eight regions of origin: 15 individuals from 15 localities and 49 variable characters. Characters contributing to each coordinate are listed in descending order, with arrows indicating the direction of increase in the value of each character (boldface characters dominate that coordinate).

Flowers of macrospecies Apifera are dominantly self-pollinating, thus encouraging the maintenance of a wide spectrum of named phenotypic variants; happily, those variants are most commonly described as infraspecific taxa rather than as different microspecies (presumably because they share the same primary pollinators, uniquely within *Ophrys*—wind and rain). Our analysis is based on 15 plants from 15 localities spanning eight geographic regions. Unusually, the first and third coordinates (Figure 14) provided a more informative plot than the first and second coordinates. Multiple samples from the same geographical region are reliably placed fairly close together.

Characters contributing to the first coordinate show that the British and Tuscan plants had sepals that were significantly smaller than those of plants from the remaining regions, but with lateral petals that were slightly longer and labella that were less hirsute. The second coordinate was dominated by subtle differences in the degree of recurvation of the petals, together with the more extensive speculum shown by the single plant sampled in northern Greece. The third coordinate was polarised between single plants from Tuscany and Epirus toward the negative end of the coordinate and from Cyprus toward the positive end, the former having toothed petals and minimal appendices, and the latter featuring lateral sepals that were oriented slightly forwards rather than being swept back in the posture more typical of macrospecies Apifera. The fourth coordinate, dominated by flower number, offered no obvious discrimination.

#### 3.4.4. Macrospecies Insectifera

**Figure 15:**
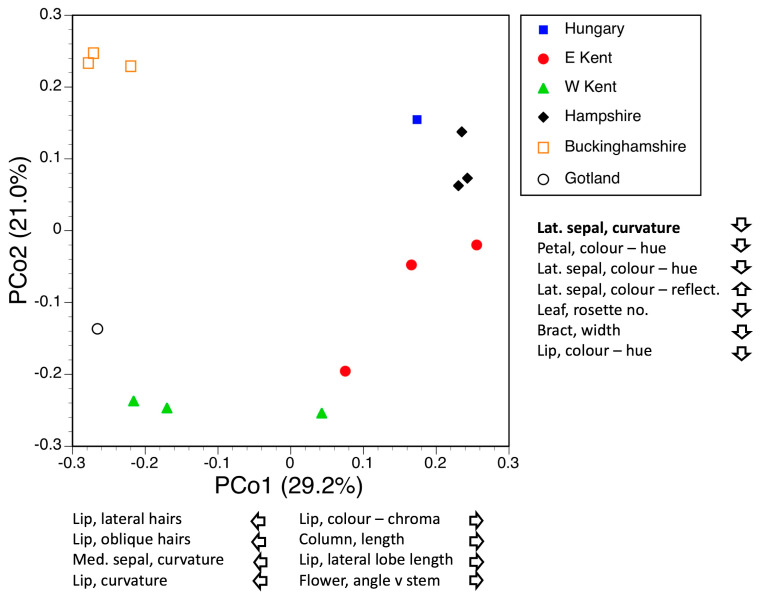
Plot of principal coordinates 1 and 2 for the macrospecies Insectifera, labelled according to six regions of origin: 14 individuals from six localities and 40 variable characters. Characters contributing to each coordinate are listed in descending order, with arrows indicating the direction of increase in the value of each character (boldface characters dominate that coordinate).

Sampling of macrospecies Insectifera was especially poor; single plants from Hungary and the Swedish island of Gotland were compared with trios of plants sampled from each of four localities that together formed a west–east transect across southern England. Multiple plants from single localities group together on the plot of the first two coordinates (Figure 15). Plants scoring negative values on the first coordinate (including Buckinghamshire and Gotland) possess discernible trichomes; their labella are moderately laterally recurved, and their median sepals are oriented vertically rather than forwards; the Gotland plant also had unusually small lateral labellar lobes. The second coordinate separated populations primarily according to lateral sepal curvature and colour, plants from Buckinghamshire and Hungary having somewhat paler green sepals that curve slightly forwards rather than being held vertically.

The third coordinate showed the non-British (i.e., Hungarian and Swedish) plants to have slightly paler petals and a comparatively shallow apical notch in the labellum. The fourth coordinate identified the Hungarian plant as having somewhat paler sepals and a narrower column; it also possessed the smallest labellum.

### 3.5. Deeper Analysis of Macrospecies Fuciflora

The above descriptions of the principal coordinates plots resulting from analyses of single macrospecies are qualitative and so vulnerable to accusations of subjectivity. In an attempt to explore a selected macrospecies more deeply and also to consider the microspecies level as seriously as the mesospecies level, we quantified the morphospace of both several mesospecies and several microspecies within the macrospecies Fuciflora—a taxon chosen because herein it is the most intensively sampled macrospecies in terms of plants analysed, is rich in microspecies and proved to be the only macrospecies wherein both the third and fourth principal coordinates appeared to offer as much taxonomic discrimination as the first two.

**Figure 16:**
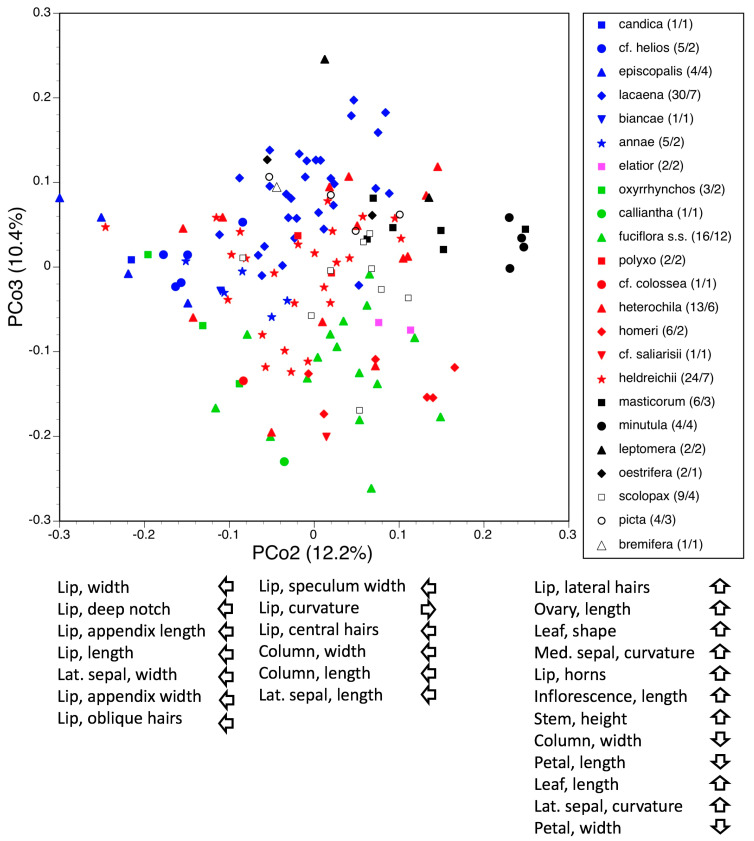
Plot of principal coordinates 2 and 3 for the macrospecies Fuciflora, labelled according to 23 microspecies (and six mesospecies, represented by identically coloured symbols): 143 individuals from 68 localities and 51 variable characters. Parenthetic numbers in the taxonomic key indicate the number of plants and localities respectively included in the analysis. Characters contributing to each coordinate are listed in descending order, with arrows indicating the direction of increase in the value of each character.

The approach taken was to quantify the morphospace occupied by each mesospecies and microspecies on four planes that each represented different pairwise combinations of the first four coordinates. For each such plane, the areal extent (i.e., morphospace) of the single macrospecies, its component mesospecies and their component microspecies were simply calculated as a convex hull (given a set of points distributed across a single plane, the convex hull of the set is the smallest convex polygon that contains all of its component points). The morphospace of each mesospecies and microspecies was then expressed as a percentage of that occupied by the entire macrospecies. Comparison was limited to those nine of the 23 sampled microspecies that were represented by five or more individuals and to those five of the six sampled mesospecies that were represented by more than 15 individuals. An exemplar plot, featuring the second and third coordinates and showing plants resolved at the microspecies level, is given as Figure 16, and the vital statistics of the four planes under comparison are given in Table 2.

Focusing initially on the plane constructed of the first and second coordinates, the average microspecies occupies 13%, and the average mesospecies 41%, of the morphospace occupied by the entire macrospecies (Table 2). However, these average figures disguise the facts that (a) there is a ten-fold difference between the most compact and most diffuse microspecies, and (b) there is a three-fold difference between the most compact and most diffuse mesospecies. Morphometric variation was greatest in mesospecies *Fuciflora*, at 67% of the total observed for macrospecies Fuciflora. We anticipated from first principles that we would find a strong positive correlation between morphospace occupied and sampling intensity, irrespective of whether this was assessed as the number of individuals or number of populations sampled per taxon. Surprisingly, for a plane constructed using the first two coordinates, this expectation was fulfilled for microspecies but not for mesospecies.

Comparison of the four planes analysed revealed both surprising trends and a surprising lack of trends, depending on the precise question being asked. The average morphospace occupied by a particular microspecies varied remarkably little between plots. Perhaps because it was already poor when viewed at the microspecies level and abysmal when viewed at the mesospecies level, the cohesion of taxonomic groups did not decrease in plots employing lower-level coordinates. Indeed, there was a modest decrease in the average morphospace occupied by mesospecies in those plots that lacked the first coordinate (Table 2)—in other words, a slight increase in their perceived cohesion.

**Figure 17:**
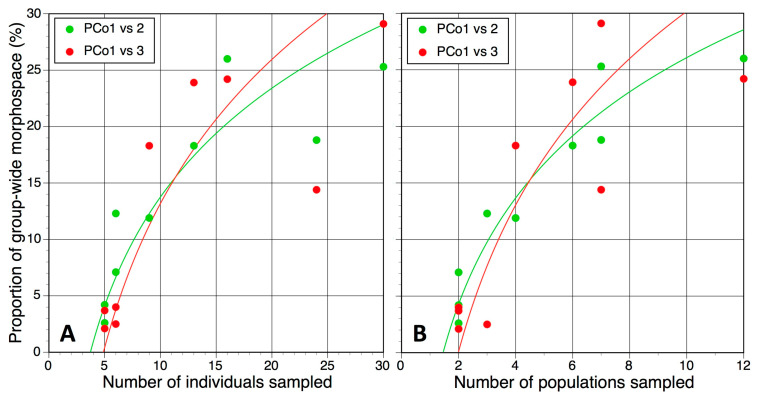
Relationship between the morphospace occupied by the nine best-sampled microspecies of macrospecies Fuciflora versus sampling effort, as measured in numbers of individual plants (**A**) and source populations (**B**) and compared for two morphometric planes (PCo1 vs. 2 and 1 vs. 3: see also Table 2).

The most obvious explanation for the radical differences in the proportion of total morphospace occupied by individual microspecies and mesospecies is the equally radical contrasts in sample size. First principles correctly predicted that curve-fitting morphospace against sample size would approximate a negative exponential curve (Figure 17). This assumption proved to be correct, though regressing morphospace against sample size for microspecies yielded moderately contrasting r^2^ values among plots—these values ranged from 0.82 for individuals and 0.92 for populations on the plot of the first and second coordinates, through to 0.53 for individuals and 0.67 for populations on the corresponding plot derived from the second and third coordinates (Table 2, Figure 17). The mesospecies deviating most from the negative exponential curves was *Heldreichii*, which offered unusually tight cohesion relative to sample size. The availability for comparison of only five mesospecies meant that r^2^ values were statistically unreliable at this higher taxonomic level but nonetheless indicated that the relationship between sample size and morphological variation of mesospecies was particularly poor for the plot of the first two coordinates, irrespective of whether sample size was represented by the number of plants or number of populations. Thus, the combination of the first two coordinates yielded the strongest positive correlations between morphological variation and sample size at the level of microspecies but apparently the weakest at the level of mesospecies, hinting that different suites of characters may experience maximum variation at contrasting demographic levels.

## 4. Discussion

### 4.1. Strengths and Weaknesses of Our Chosen Morphometric Approach: General Principles

Before drawing specific conclusions regarding phenotypic variation across the genus *Ophrys*, we will first critically appraise the morphometric approach that we have employed here, in order to establish limits to the strength of the conclusions that can reasonably be drawn from this study. We should begin by noting that this is the 22nd such empirical study of European native orchids that one of us (R.B.) has published using this morphometric approach, together spanning seven genera: *Dactylorhiza* (6 papers [66,77]), *Gymnadenia* (2 papers [69,78]), *Platanthera* (4 papers [79,80]), *Pseudorchis* (1 paper), *Orchis* (4 papers [76,81]), *Himantoglossum* (3 papers [82]) and *Ophrys* (1 paper [45]). The strengths and weaknesses of our approach have therefore been amply demonstrated empirically.

Our objective is to characterise rapidly but accurately the macromorphology and ‘mesomorphology’ of the above-ground organs of each plant studied through minimal intervention by leaving that plant in situ and removing only one or two flowers for later morphological and possibly DNA-based examination. This approach is intentionally low-tech, requiring only a ×8/×10 loupe, an RHS colour chart and a well-designed proforma data-sheet. Once placed under such close scrutiny, a terrestrial orchid genus can typically be resolved into between 40 and 55 credible numerical characters, the total depending on its relative morphological complexity. The initial aim is to characterise all above-ground organs with similar levels of resolution, though this principle does not yield similar numbers of characters per organ; the greater complexity of the flowers in general and labellum in particular inevitably means that they are divisible into larger numbers of valid characters than are the vegetative organs. In order to employ the widest feasible range of characters representing, at contrasting scales, size, shape, texture and colour, it is necessary to establish a mixture of characters that are genuinely quantitative (i.e., metric measurements and meristic counts) alongside characters that are semi-quantitative (i.e., multistate and bistate), in some cases requiring the imposition of potentially arbitrary state boundaries on variables that are arguably better described as qualitative.

Dividing organs into characters (and, in the case of semi-quantitative characters, dividing each such character into a linear sequence of alternative character states) requires much decision making that can only realistically be conducted under self-imposed prior constraints. These are, of necessity, guidelines, as attempts to develop fixed unbreakable rules generally prove counter-productive. Many of the most difficult decisions faced during this process concern maintaining a balance between the desire to maximise overall character number versus the desire to avoid duplications of similar characters that will likely cause spurious positive correlations—correlations capable of dominating the ensuing algorithmic analyses (see examples in *Ophrys* discussed below in Section 4.2). Also challenging is choosing the optimal number of character states to represent semi-quantitative characters such as the shapes of particular organs or features. Increasing the number of states per character tends to decrease the influence of the relevant feature on the subsequent analysis, but conversely, recognising too few states risks over-weighting a boundary between states that proves to be arbitrary.

One issue that requires post-hoc resolution is that, given the many life-challenges experienced by a typical plant, the completed morphological matrix for a large number of study plants will contain at least a small proportion of missing values, representing characters that could not be scored for a minority of individuals. Possible causes of missing values include damage to that feature of the plant (e.g., through herbivory or even carelessness on the part of the analyst) or programmed/environmentally induced decay prior to sampling (e.g., death through desiccation of the basal rosette of leaves prior to anthesis). Although such undesirably empty matrix cells can be filled through the application of arbitrary rules (for example, simply by inserting the group mean value), obviously it is better if the chosen analytical algorithm can instead readily accommodate missing values.

Popular multivariate algorithms such as principal components analysis (PCA) are designed to analyse matrices that are both complete and that consist of homogenous, fully quantitative characters. In contrast, we exclusively employ principal coordinates analysis (PCoA) based on the Gower similarity coefficient [73,74] because this approach (a) successfully accommodates heterogeneous suites of both fully quantitative and semi-quantitative characters, and (b) yields results that are unaffected by missing values. The price paid for these clear advantages is that it is more difficult to ascertain the relative contributions of the original characters to the resulting trees or ordinations; fortunately, this goal can be achieved in Genstat [70] via pseudo-F statistics derived through the ‘RELATE’ command. The most obvious alternative to PCA and PCoA is canonical variates analysis (CVA), but in our opinion, this approach is unnecessarily subjective, at least when attempting circumscription—it requires that the algorithm should be informed a priori of the assignment of individuals to groups and then maximises perceived distances between groups at the expense of distances within them.

Once the similarities among the study plants have been quantified, the choice of presentational style also has a profound effect on the resulting interpretations. Three options merit brief comment here: rooted dendrograms, unrooted minimum spanning trees (both calculated directly from symmetrical matrices of the Gower similarity values and so summarising all the data input) and principal coordinates ordinations, which abstract from the data those planes that encompass relatively high variation and so effectively employ only a (calculable) proportion of the total variability. Interestingly, experience has taught us that, for morphological data, ordinations are consistently more congruent with prior taxonomy than are either unrooted trees or, especially, rooted trees. A single “errant” maximum similarity value can cause major distortions to relationships inferred among plants placed distally to the suspect maximum similarity value within a tree, whereas such “errors” are in practice more buffered within ordinations, wherein they can at worst only influence the spatial placement of the “errant” individual itself.

In addition, a tree is a singular result that must be either accepted or rejected by the analyst, whereas several principal coordinates can readily be plotted in various combinations, allowing the analyst to search for the most informative plane(s) of variation. Admittedly, as demonstrated here, valuable information is rarely obtained from coordinates of lower order than the third. Superimposing a minimum spanning network onto a principal coordinates plot can, in some cases, usefully assist interpretation, though this approach becomes impractical to draft for presentation in ordinations involving large numbers of points; it is most effective when applied to plots ordinating mean values for populations [69,77,82], rather than to plots based on raw data for individual plants, such as those presented herein in Figure 5, Figure 6, Figure 7, Figure 8, Figure 9, Figure 10, Figure 11, Figure 12, Figure 13, Figure 14, Figure 15 and Figure 16.

### 4.2. Strengths and Weaknesses of Our Chosen Morphometric Approach as Applied to Ophrys

Our standard sampling strategy for morphometric studies focuses on the principle of reciprocal illumination among three contrasting demographic levels: individual plants, local populations, and aggregates of populations that have the potential to be circumscribed as species (or, failing that, as infraspecific taxa). The amount of research time invested in such a study therefore depends on the number of characters measured and relative numbers of putative species, populations per putative species and plants per population that are scored, based on a carefully planned and geographically extensive sampling strategy [29,67,68]. Typical figures for these parameters in our previous morphometric studies have been 2–10 putative species, 5–20 populations per species and 10(–20) plants per population [66,69,82,83]. Unfortunately, it was impractical to bring an equivalent level of sampling rigour to the present study, given that the primary objective was to characterise the broader morphological trends across a genus considered by some specialists to contain approximately 400 (micro)species. The 457 plants sampled for the present study collectively represent 113 microspecies (Table 1), a ratio of individuals per putative taxon that renders the representation of most microspecies perilously close to typological. It therefore became necessary to accept as fixed a priori both the microspecies, previously circumscribed through traditional morphology, and the macrospecies, previously circumscribed through several DNA-based studies culminating in next-generation DNA data [43]. Our primary objective was to explore whether credible circumscriptions can be achieved at the intermediate demographic level of mesospecies [45], beginning with those designated through several iterations by Delforge [38,39].

When designing the present study, we considered the possibility of characterising microscopically the labellum of each measured plant, but time constraints encouraged us to settle for the more typological approach of placing the columns of representative individuals of a few microspecies under the scanning electron microscope (Figure 4). In retrospect, our study would certainly have benefited from the inclusion of an additional phase of data collection conducted under a binocular microscope in order to better detail micromorphological features of the gynostemium, stigmatic surface and proximal labellar ‘neck’ (such as the paired ‘pseudo-eyes’ that characterise some groups within *Ophrys*: [12,13,37,43]). Although the present total of 51 scored characters falls well within our previously accepted range of 40–55 characters, it was inevitable that some characters would became inapplicable or invariant in some of the present subordinate analyses that are based on only a single macrospecies; predictably, this phenomenon peaked (at 11 uninformative characters) when analysing the morphologically least complex macrospecies, Insectifera.

In previous morphometric studies of orchid genera such as *Dactylorhiza* [66,77] and *Platanthera* [79,80,83], we have found vegetative characters to be relatively problematic in that they are on average considerably more variable than floral characters [76], collectively reflecting the vigour of the plant in question. The life history of most terrestrial orchids involves the annual replacement of their all-important stem-tuber; the relative success of this process of somatic replacement strongly influences the likelihood that a plant will flower during the following spring/summer and, if so, how vigorously. Obviously, vegetative vigour has a strong underlying genetic influence and so can genuinely reflect taxonomic differences, but it is also strongly influenced by the environment in which the plant is growing—not only in the present growing season but also in the previous growing season. It becomes difficult—arguably impossible—to disentangle genetic from ecophenotypic factors influencing plant size. In contrast, ecophenotypic influences have a much weaker impact on most floral characters because flowers emerge over a much shorter time period and are generated under stronger developmental constraints. It is therefore helpful, at least from this perspective, that vegetative characters have been proven to exert only modest influence on most of the ordinations conducted for the present study. This observation suggests that the ratio between degrees of vegetative variation and floral variation is lower in *Ophrys* than in comparable orchid genera (see Section 4.4), as well as indicating that variation is poorly correlated between these two functional categories of organ.

In the case of *Ophrys*, it is among the flowers rather than the vegetative organs that concerns are raised regarding positive correlation among potentially co-functional characters. Arguably the most extreme example is provided by our decision to quantify the background colours of three different floral organs: the labellum, lateral petals and lateral sepals. Representing colour through the CIE system required us to report each colour as three characters (chroma, hue, reflectivity), thereby causing flower colour to dictate a total of nine of the 51 morphometric characters scored. In fact, little variation was detected in labellum colour, but inevitably, similarities in colour between the lateral petals and lateral sepals were evident in many plants. On the other hand, colour differences between petals and sepals have been judged taxonomically diagnostic among *Ophrys* microspecies within some macrospecies. Although there are no definitively right or wrong answers when addressing such conundra, it is important to understand the potential consequences of such decision-making for the patterns seen in the resulting ordinations.

It is also worth considering the likely morphological transitions that dictated the origin of the genus *Ophrys*. All genera closely related to *Ophrys* [20,84] have labella that are largely two-dimensional, the third dimension being confined to the simple curvature of the lateral margins, except that, proximally, the labella of genera such as *Neotinea*, *Himantoglossum* and *Anacamptis* are consistently invaginated into an elongate cylindrical spur that may or may not secrete nectar [85]. We suspect that a key stage in the emergence and initial diversification of the genus *Ophrys* was the evolutionary loss of that spur, because it liberated the labellum to develop much more complex three-dimensional topographies—topographies that become evident only after the labellum inverted from concave to convex during the opening of the bud [3] (Figure 2 and Figure 3). There is a general trend for labellum topography to be both more three-dimensional and more complex in the later-diverging macrospecies of *Ophrys*, contrasting especially sharply with the comparative simplicity of the early diverging Insectifera [12,43].

Some recent studies of European orchid clades have employed landmark analysis of labella as a time-saving proxy for all morphological variation within the study group [86,87,88], but this approach is less utilitarian than attempting to comprehensively quantify the entire above-ground portion of the plant. Firstly, it provides little useful information toward improving previous formal taxonomic descriptions of the taxon. Secondly, much controversy still surrounds the relative contributions to successful pollination of labellum topography compared with labellum surface texture, labellum markings, tepal colour, overall flower size and especially the complex biochemistry of volatiles exuded by the flower (features considered in greater detail in Section 4.5). Just which properties qualify labellar topography to be given pride of place in this spectacular phenotypic pantheon?

One obvious question to ask at this point is whether our 51 morphometric characters are collectively as effective as the human eye when seeking morphological differences among *Ophrys* plants? The answer is almost certainly no; the same eye–brain coordination that allows us to discern among innumerable human faces is likely to be almost as adept in distinguishing among a panoply of bee orchid flowers. The advantage of the detailed morphometric approach is that the principal coordinates algorithm is fastidiously objective; it has no prior expectations of groupings, nor is it determinedly seeking minute differences while overlooking numerous similarities.

In summary, despite the panoply of “Faustian bargains” that we found to be necessary in order to bring to eventual fruition this challenging morphometric study, we are confident that the collective ordinations presented here as Figure 5, Figure 6, Figure 7, Figure 8, Figure 9, Figure 10, Figure 11, Figure 12, Figure 13, Figure 14, Figure 15 and Figure 16 provide a reasonably accurate picture of not only any broad morphological trends that are evident across the genus *Ophrys* as a whole but also any trends that dominate within each of its nine macrospecies. We will first attempt to summarise those trends and then consider their implications for both the taxonomy and evolutionary mechanisms inferred within this highly contentious ‘model’ genus.

### 4.3. Overview of Morphological Variation within Ophrys

#### 4.3.1. The Search for Discontinuities among Taxa

We hoped, rather than confidently expected, to detect morphological discontinuities among at least the *Ophrys* macrospecies [28,45]. Ideally, they would coincide with consistent discontinuities identified in molecular data. However, among the 11 ordinations performed by us that employed suites of characters that were both complete and unmodified (Figure 5, Figure 6, Figure 7, Figure 8, Figure 9, Figure 10, Figure 11, Figure 12, Figure 13, Figure 14, Figure 15 and Figure 16), only two—each confined to a single-macrospecies—yielded clustering of taxa that was sufficiently cohesive to separate those taxa by apparent morphological discontinuities. The first such case was the ordination of macrospecies Speculum (Figure 11), which readily distinguished the only two microspecies included in the analysis, thereby implying that *O. speculum* s.s. and *O. regis-ferdinandii* are separated by a morphological discontinuity. However, it is likely that this distinction would have been weakened had we included in the analysis the morphologically intermediate microspecies from Iberia, *O. vernixia* [39].

The second discontinuity was observed within macrospecies Umbilicata (Figure 9), separating the Cypriot microspecies *levantina* and *aphrodite* from the remaining six microspecies analysed here, which have labella that are far more three-dimensional and bear specula that are both more complex and more striking. We suspect that this apparent discontinuity would, uniquely, survive the sampling of all of the microspecies attributed to macrospecies Umbilicata. This is the only credible example that we detected of a modest morphological discontinuity occurring within a single genetically cohesive macrospecies. Otherwise, the clear impression gained at both the macrospecies and mesospecies levels was of a morphological continuum rather than an aggregate of discrete, readily identifiable taxa.

#### 4.3.2. The Search for Trends in Character Correlation

The simultaneous multivariate analysis of the entire genus *Ophrys* (Figure 5) is dominated by some suites of characters that also dominate the majority of single-macrospecies analyses (sepal and lateral petal colour, labellum dimensions), some suites of characters found in only a minority of single-macrospecies analyses (presence and size of a labellar appendix and of the associated apical notch, labellar trichomes) and one character that dominates no other analysis (pale labellar margin). It is challenging to identify any generalised patterns of character variation by comparing the series of analyses of single macrospecies.

Understandably, the colour of sepals and lateral petals contributes most to analyses of macrospecies that contain a mixture of green-sepaled and pink/purple-sepaled plants (Fuciflora, Umbilicata, and Sphegodes, wherein the presence or absence of sepal-stripes also contributes), but flower colour variables also contribute to some macrospecies that maintain contrasting shades of green (e.g., Bombyliflora and Insectifera). Sphegodes and Fuciflora are unusual among macrospecies in that speculum characters contribute comparatively weakly. Plant size plays a greater role within some macrospecies than others, but no overall pattern can be discerned; overall, vegetative characters are subordinate to floral characters. Much to our surprise, sepal dimensions dominate over the dimensions of lateral petals, labella and gynostemia, which contribute substantially to ordinations mainly in macrospecies that are relatively poor in both microspecies and mesospecies—a statement that also applies to lip shape/lobing and trichome distribution, as well as median sepal shape. When seeking generalisations, the main conclusion to be drawn from Figure 5, Figure 6, Figure 7, Figure 8, Figure 9, Figure 10, Figure 11, Figure 12, Figure 13, Figure 14, Figure 15 and Figure 16 is simply that morphological variation within the genus is exceptionally multidimensional.

#### 4.3.3. Residual Incongruence between Macrospecies and Mesospecies

The four editions of the traditional monograph by Delforge [38,39] have gradually and partially converged on the results of several 21st century molecular studies that together have provided strong evidence for circumscribing nine macrospecies; consequently, each of the 29 mesospecies can now largely be accommodated (often in multiples) within a particular macrospecies. The major exception to this generalisation concerns Delforge’s mesospecies *Bornmuelleri*, which substantially transgresses the boundary separating macrospecies Fuciflora from macrospecies Umbilicata (Figure 1). RAD-seq, nrITS and plastid sequences have all shown conclusively that the Levantine microspecies *bornmuelleri* and *levantina* (presumably also the morphologically very similar *aphrodite* and *carduchorum* [39]) belong to macrospecies Umbilicata [21,43], in which they form a monophyletic pairing that diverged after *attica* and *kotschyi* but before *umbilicata* s.s. and its close relatives [G. Sramkó, O. Paun and R. Bateman, unpublished data]. In contrast, other microspecies assigned by Delforge to his mesospecies *Bornmuelleri*, such as *episcopalis*, *aeoli*, *candica* and *biancae*, are placed molecularly within macrospecies Fuciflora [21,43,44]. For example, RAD-seq data generated by Sramkó, Paun and Bateman [unpublished data] nest *biancae* within a tight-knit clade of samples from southern Italy that also consists of the microspecies *lacaitae*, *oxyrrhynchos* and *celiensis*, all three of which were placed by Delforge [39] in his mesospecies *Fuciflora*.

As demonstrated here, the morphologies of microspecies such as *bornmuelleri*, *levantina* and *aphrodite* deviate considerably from those of other members of macrospecies Umbilicata (Figure 9). Even in the principal coordinates plot encompassing all macrospecies (Figure 5), *levantina* and *aphrodite* did not overlap with the remainder of macrospecies Umbilicata, but neither did they overlap substantially with macrospecies Fuciflora; instead, values of between –0.03 and –0.16 for the second coordinate placed plants of these taxa mid-way between the rest of macrospecies Umbilicata and macrospecies Tenthredinifera on Figure 5. Their molecular phylogenetic placement (Figure 1) suggests strongly that characters causing them to more closely resemble macrospecies Tenthredinifera and some members of macrospecies Fuciflora—their less laterally recurved labellum with a uniformly villose margin, relatively small, simple speculum and relatively large appendix—arose independently. Thus, although their morphological distinctiveness is arguably sufficient to justify Delforge’s [38] decision to separate these microspecies as a mesospecies outside the Umbilicata group, mesospecies *Bornmuelleri* as currently conceived by Delforge is not a natural grouping and therefore requires re-circumscription.

The empirical aims of the present study are too modest to encourage us to meddle below the macrospecies level in previous classifications of the family. Our taxonomic principles rely upon the ability to seek congruent discontinuities in multiple datasets, but herein we are reporting on only one (albeit large) new dataset that representing morphometrics. Both species concepts and how they are applied require further clarification if genuine progress is to be made.

### 4.4. Comparison with Morphological Variation Observed in Other European Orchid Genera

Characterising morphological variation in bee orchids as near-continuous and exceptionally multidimensional encourages us to compare our study of *Ophrys* with those conducted by us previously on other genera of European orchids [45,66,69,76,77,79,80,81,82,83].

In any analysis, the percentage of variance accounted for by the first two principal coordinates provides a useful indication of the relative degrees of dimensionality in the underlying morphometric data. The higher the dimensionality, the more difficult it becomes to partition individuals and populations into well-circumscribed taxa. Here, the analysis that included all nine macrospecies of *Ophrys* (Figure 5) accounted for 34% of the total variance, a figure consistent with most of the remaining analyses involving subsets of the master data. Most plots occupied the range of 30 to 34%, falling to a nadir of 27% in the analysis of macrospecies Fuciflora (Figure 8). Predictably, morphometric dimensionality was lower in three of the four analyses that encompassed only a small number of plants representing just one or two microspecies (macrospecies Speculum, Apifera and Insectifera), in which the total variance increased to 46–50%. A further indication of comparatively high dimensionality in the majority of ordinations was a small difference for separating the first and second coordinates in terms of the amount of variance that each coordinate accounted for—less than 3% in the case of the single-macrospecies analyses conducted on the Sphegodes and Fuciflora groups (Figure 7 and Figure 8).

Comparison with the vital statistics of 22 in situ morphometric surveys previously conducted by us, spanning seven genera of European orchids, showed that the proportion of the total variation encompassed by the first two principal coordinates was weakly negatively correlated with both the number of plants analysed in the study and the number of characters that could usefully be scored for that particular genus. This conclusion is unsurprising, as increasing the number of plants analysed or characters scored inevitably leads to at least modest increase in the spectrum of variation captured during sampling. Nonetheless, the morphological distinctiveness of species in the genus in question was the primary factor that dictated the amount of variation recovered in that first multivariate plane defined by coordinates 1 and 2.

European *Platanthera* species show comparatively little variation in floral shapes and colours, differing mainly in the relative size of various floral organs; consequently, each species forms a discrete cluster [79,80,83]. In common with studies of hybrids between two morphologically distinct parents [89], they yield highly polarised plots of PCo1 versus PCo2 that encompass approximately 70% of the total variation. In addition, much of the variation tends to be encompassed by the first coordinate, creating an unusually large differential between the first coordinate and the second. Studies of genera that contain a mixture of species separated by morphological discontinuities and species possessing overlapping morphologies, such as *Himantoglossum* [6,82] and the anthropomorphic subgenus of *Orchis* [76,81,90], capture 40–50% of the total variance in the first two coordinates. But taxonomic groups in which none of the species are separated by morphological discontinuities yield plots that capture only approximately 30% of the total variation in the first two coordinates. Examples include the *Dactylorhiza majalis* aggregate [66,91], in which species have originated from repeated allopolyploidy (hybridisation combined with genome doubling) events between different ecotypes of the same two parental lineages [92,93,94], and the *Gymnadenia conopsea* aggregate [69,78], in which three species have diverged substantially in DNA barcode regions, flowering times and preferred habitats but only subtly and unreliably in morphology [69,95].

Morphometric analyses of *Ophrys* [45], notably those presented here, make clear that patterns of morphological variation within this entire genus are comparable with those observed in the *Dactylorhiza majalis* and *Gymnadenia conopsea* aggregates alone. Although there remain vigorous debates regarding the choice of species versus subspecies status for taxa within both the *D. majalis* [36,91] and *G. conopsea* [78,96] aggregates, there is greater consensus regarding the number of such taxa in each: *D. majalis* s.l. encompasses about ten species/subspecies, whereas *G. conopsea* s.l. encompasses only three or four. These figures present a radical contrast with estimates of up to 400 microspecies in the genus *Ophrys* and at least 113 microspecies in just one of the molecularly circumscribed macrospecies, Sphegodes [39,45].

Given (a) the demonstrable morphological overlap of *Ophrys* taxa at all three analytical levels (i.e., macrospecies, mesospecies and microspecies), (b) the multidimensionality of that variation and limited correlation among morphological characters and (c) the genetic overlap that has been repeatedly demonstrated at the mesospecies and microspecies levels [21,43,44,45], can a single morphological continuum lacking directional evolutionary trends justifiably be claimed to encompass up to 400 species? To address that question, we need to move beyond merely describing variation in order to consider the mechanisms likely to have generated that variation.

### 4.5. Review of Features Encouraging the Three Phases of Pseudo-Copulatory Pollination

The attraction of naïve male hymenopterans to interact with their flowers, using the sequential olfactory, visual and tactile cues, has long been the cornerstone of bee orchid studies. Herein, we briefly consider the nature of those three cues, addressed in reverse sequence.

#### 4.5.1. Tactile Stimuli

Setting aside very few exceptions, including the comparative SEM images of gynostemia illustrated here (Figure 4), detailed morphological studies of *Ophrys* have focused almost exclusively on describing the labellum. This emphasis is understandable, given its often rugged three-dimensional topology [22,87]; the complexity of its surface textures and markings [97]; and its equally complex internal anatomy [12,13,14,98]. Even genome size differs among cells within different regions of the labellum, presumably dictating contrasting levels of glandular activity [14]. The labellum undeniably gives every appearance of representing an exceptional aggregate of numerous adaptations, all finely tooled by natural selection to seduce a particular (range of) species of pollinating insect into repeatedly attempting copulation.

However, most accounts of the functional morphology of the labellum remain largely anecdotal. Interpretations have focused on two main issues: (a) the spatial fit of the visiting male insect to the labellum in terms of size and shape, and (b) putative labellar adaptations, especially broadly concentric markings (Figure 2A) and differential expression of surface features such as trichomes (Figure 2A and Figure 3), that encourage the insect to adopt the optimal orientation for collecting pollinaria on its head (most species) or its abdomen (most commonly members of macrospecies Fusca). Variation in the size, density and orientation of trichomes is especially well illustrated in the supposedly abdominally pollinated *O. pallida* featured in Figure 3A–D. SEM imaging was used by Cortis et al. [99] to compare trichomes in the localised labellar regions of two microspecies of macrospecies Fusca on Sardinia.

Rakosy et al. [100] observed the behaviour of bees visiting flowers of *O. leochroma* (macrospecies Tenthredinifera) from which contrasting segments of the labellum had been removed, noting that the removal of those portions used more frequently by the bees as gripping or contact points caused greater reductions in the frequency and effectiveness of pollination, especially in the deposition of pollinaria. These observations led the authors to predict that those regions most important in ensuring mechanical fit between flower and pollinator—in this case, the stigma and “shoulders/horns” of the labellum—would operate under strong stabilising selection within this microspecies, whereas regions such as lateral lobes, appendix and associated apical notch would be less critical and would therefore show greater variation. We applaud such experimental approaches and wonder whether “shaving” critical regions to remove their trichomes might enable a more subtle approach to exploring the relative importance (or otherwise) of tactile stimuli in encouraging successful pollination?

#### 4.5.2. Visual Stimuli: Significance (or Otherwise) of Sepal Colour

Compared with the large number of investigations of *Ophrys* pseudo-pheromones, visual cues such as flower colour have been under-researched. The most notable exception is the series of behavioural experiments conducted by Hannes Paulus and colleagues using the pink-sepaled microspecies *heldreichii* (macrospecies Fuciflora) as their experimental system. Initially, Spaethe et al. [101] recorded the effect on pollinator attraction of removing the sepals and lateral petals. When tested using the eucerid bee *Tetralonia* (*Eucera*) *berlandi*, excision reduced the attractiveness of an actual flower by ca. 50% and that of an imaged flower by ca. 90%, once the insect was within 30 cm distance of the flower [102]. These results were broadly supported by later field testing, which also confirmed low figures for average male (ca. 8%) and female (ca. 4%) reproductive success of intact *heldreichii* flowers, figures typical of the genus [103].

Streinzer et al. [104] then compared microspecies *heldreichii* with the morphologically similar but phylogenetically distant microspecies *dictynnae* (macrospecies Tenthredinifera), which has sepals of a paler pink and “no conspicuous labellar pattern” (better described as a less complex and less extensive speculum; it is actually of roughly equal brightness to that of *heldreichii*). Their results suggested that the prominence of the speculum had little effect on pollinator preference but that having paler pink sepals, closer to medium green hues in green-receptor reflectance, reduced male pollinator preference by ca. 70% [104]. In contrast, Vereecken and Schiestl [105] found no behavioural difference in pollinators presented with a green versus ‘white’ sepal polymorphism in *O. arachnitiformis* (macrospecies Sphegodes).

In this context, we note that, for humans, white is not a true colour but rather an absence of colour, though this statement does not apply to the contrasting visual spectrum of insects [106]. In any case, once they had been colour-matched by us, supposedly white *Ophrys* sepals prove to be either pale green or pale pink. Moreover, the contrasting colours are located in different tissues within the perianth; pink–purple anthocyanins are typically diffused within the cytoplasm of epidermal cells, whereas green chlorophylls are packaged in chloroplasts that are more strongly concentrated in the underlying mesophyll [107]. Viewed from a genetic perspective, perianth colour characters appear to be not only polymorphic but also developmentally unstable, emphasised by the fact—demonstrated through captive breeding—that a single self-fertilised *Ophrys* flower can be capable of producing both green-flowered and pink-flowered progeny [108].

Our data show that two-thirds of microspecies allocated to macrospecies Sphegodes are predominantly green-sepaled rather than pink-sepaled; a literature search by Spaethe et al. [109], sampling 71 microspecies across several macrospecies, estimated a similar proportion of green-sepalled taxa. It also suggested a strong bias between those species typically pollinated by *Andrena* s.l. bees (91% green-sepaled) and those typically pollinated by the more visually acute *Eucera* s.l. bees (83% pink-sepaled).

**Figure 18:**
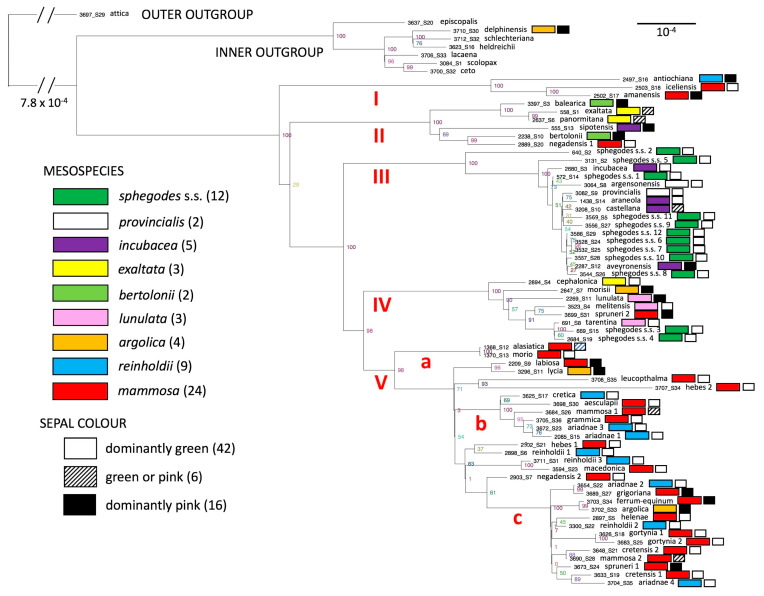
Maximum likelihood phylogeny of whole plastomes obtained via genome skimming from 64 individuals representing 40 microspecies of macrospecies Sphegodes, together with six individuals of macrospecies Fuciflora (‘inner outgroup’) and one individual of macrospecies Umbilicata (functional ‘outer outgroup’). Plants are named according to microspecies, and box-labelled according to both mesospecies attribution and the dominant colour of their sepals. Roman numerals and lower-case letters indicate the main haplotype clusters and subclusters within macrospecies Sphegodes. Figures supporting nodes are bootstrap percentages. Topology from Bateman et al. [45].

The RAD-seq network (Figure 1) implies that there were not one but two phylogenetic origins of anthocyanin in sepals and lateral petals; one origin immediately prior to the divergence of macrospecies Apifera (i.e., permeating through groups F–J) that is a synapomorphic tendency (characterising some but not all microspecies) and one origin during or after the divergence of macrospecies Tenthredinifera (group B) that is a true synapomorphy (characterising all microspecies). Similarly, the evolutionary spread of dark purplish–brown labellar pigment downwards and outwards into green lateral sepals also shows two origins in Figure 1: the pigment forms two stripes (one dorsal and one ventral) in macrospecies Speculum but develops as a single more diffuse zone (ventral only) in some but not all microspecies of mesospecies *Mammosa* and *Reinholdii* (within macrospecies Sphegodes, roughly equating with haplotype clade V in Figure 18). These patterns of phylogenetic transitions do not suggest that flower colour is of especial utility, either evolutionary or taxonomic.

Like flower colour, labellum pattern has also been subjected to experimentation. Labellum patterns are said to be learned by unsatisfied male insects in order to avoid time-wasting re-visitations of females already mated, therefore diverse speculum patterns evolved in *Ophrys* species to slow the learning process [25]. Admittedly, Stejskal et al. [97] were obliged to employ honey bees rather than the preferred pollinator of microspecies *heldreichii* in their otherwise sophisticated behavioural experiments. Evidence that the honey bees eventually learned to avoid particular speculum shapes that had previously proved unrewarding of nectar-substitute is convincing, but the learning process required at least 50 bee–flower interactions to achieve statistical significance and did not plateau until ca. 90 interactions had occurred—a process far too protracted to facilitate *Ophrys* pollination. The authors were therefore required to suggest that sex is a far stronger motivation for male–male competition than food and would encourage far faster learning, and that this process would drive negative frequency-dependent selection, further diversifying the range of specula maintained within bee orchid populations [97].

In this context, we note that our sample of 24 plants of *heldreichii* from seven populations on Crete collectively occupied a relatively small area of the overall morphospace within macrospecies Fuciflora (Figure 16). Moreover, most of these plants (83%) were scored as possessing the same (most complex) speculum category, 4: single ring with radiating projections or multiple rings (Appendix A). Rather, we detected considerably greater variation in speculum morphology in other microspecies within macrospecies Fuciflora, notably the 30 plants together representing seven populations of microspecies *lacaena* scored by us in the Peloponnese. Does this result mean that potential pollinators of macrospecies Fuciflora occurring in the Peloponnese are more skilled at pattern recognition than their equivalents in Crete? Or are we instead observing random variation of no great evolutionary consequence?

#### 4.5.3. Olfactory Stimuli

Much of the scientific research performed on selected *Ophrys* species has focused increasingly on the complex composition of their pseudo-pheromone cocktails and on determining which groups of insects are excited by which elements present in those cocktails. The relationship between C21–C29 n-alkenes and admixed n-alkanes has attracted particular attention. Some alkenes are more attractive to male than female bees and have consequently been accused of constituting a pre-adaptation that facilitated the diversification of alkenes in *Ophrys*; this in turn is considered to have driven increased reliance on pseudo-copulation for reproductive assurance [110,111]. Experiments suggest that the bee orchid’s pseudo-pheromone cocktail must accurately mimic that of the females of the pollinating species, both qualitatively and quantitatively, which raises interesting (and thus far unanswered) questions about how the transition from pre-adaptation to adaptation may have occurred. Research also revealed that “inactive” (perhaps more accurately described as ineffective) compounds in the cocktails show much greater variation among individuals than do “active” compounds and that the flowers are sufficiently rich in these compounds to routinely outcompete for male attention the females of the relevant pollinating species [16,112,113,114].

Numbers of active compounds per microspecies typically exceed 20, offering ample variation for fine-tooling contrasts among individuals in the amounts of particular components. For example, the cocktail of *O. sphegodes* s.s. is reportedly dominated by 9- and 12-alkenes, whereas that of the closely related microspecies *O. archipelagi* are dominated by 7-alkenes differing in the precise locations of double bonds [18,22,115,116]. Valiant attempts to compare relative degrees of perceived divergence in cocktail compositions, pollinator spectra and genetics have tended to be undermined by inadequate molecular phylogenies [110].

#### 4.5.4. Overview

We conclude that increasing excitement surrounding the novelty and effectiveness of the olfactory cues for pollinators have tended to eclipse research interest in the visual and tactile cues. In practice, morphology has played a largely passive role in attempts to understand the evolution and systematics of bee orchids. Most studies have been confined to just one or two microspecies previously recognised through traditional, authoritarian taxonomy, an approach lacking explicit phases of data collection and subsequent algorithmic analysis. Few populations are sampled, and few if any morphological characters are measured. Morphology has typically been a bystander in a contest for supremacy in species circumscription that has been fought between datasets based on pseudo-pheromones plus pollinator identities versus those based primarily on DNA sequences, now supplemented with the crude overview of morphology presented in this paper. These two highly conflicting species concepts merit reappraisal in the light of this and other recent studies.

### 4.6. Microspecies versus Macrospecies

#### 4.6.1. Basis of the Ethological Species Concept

The textbook pseudo-copulatory pollination syndrome of *Ophrys* has been subject to innumerable reviews, their increasing sophistication providing a testament to the progressive accumulation of supporting evidence [25,26,31,40,42,117,118,119,120,121,122,123]. The most detailed recent overview was provided by Baguette et al. [25], who argued for a mutually beneficial co-evolutionary relationship that is asymmetric—essential for the survival of the orchid but not for that of the insect partner. The scenario requires four predicates:

(1) Male pollinators (most commonly solitary bees) emerge before females, are more likely than females to disperse beyond their foraging range, and are hard-wired to recognise and avoid both kin and non-receptive females.
(2) There exists strong infra-specific competition among *Ophrys* individuals for the attraction of species-specific hymenopteran pollinators. The remarkable learning and memory abilities shown by bees in particular induce strong selection pressure for increased variation, especially in pseudo-pheromone bouquets. The novel orchid bouquets are best regarded as evolutionary darts, thrown randomly at a dartboard of many novel potential pollinators co-occurring with the orchid, in the hope of proving adaptive by securing a reasonable match with a female insect’s bouquet and thus initiating a pollinator shift into a supposed pollinator-free space.
(3) Once the orchid has fortuitously generated an effective bouquet, directional selection then reinforces the attraction of the newly acquired pollinator by adaptively refining the morphology and/or phenology of the flower to better suit the pollinator.
(4) Male members of the pollinating species then increasingly rely on their co-evolving *Ophrys* species to locate, from a distance, suitable habitats within ecologically complex landscapes, thereby increasing the probability that they will encounter a receptive female. Once males have successfully memorised the local diversity of pseudo-pheromone bouquets (and been repulsed by post-pollination alterations in the bouquets), they are more likely to disperse to other suitable habitats, thereby reducing the frequency of self-fertilisation in both the orchid species and its pollinator.

Admittedly, given that (a) bees are vagile whereas orchids are sessile, (b) the decision where to lay her eggs rests with the female bee, and (c) females of any animal species are judged by us to be inherently more intelligent than the equivalent males, one might have predicted from first principles that female solitary bees would instead elect to nest well away from bee orchid populations, in order to avoid unwelcome competition from flowers better-endowed with pseudo-pheromones than their intended paramours [124].

Derived from this widely accepted understanding of the evolutionary process, the ethological species concept relies on pollinator-limited reproduction and consequently reaches its strongest development in cases of the comparatively inefficient pseudo-copulatory mechanisms. At its core is the reportedly intimate relationship between a particular insect species observed to pollinate the flowers and a particular comparable cocktail of pseudo-pheromones in the orchid flower, supported to a lesser degree by an appropriate size and three-dimensional shape and texture of the labellum (and gynostemium). Most studies providing data in support of this species concept were designed primarily to better understand this evolutionary mechanism rather than to circumscribe species (or, more likely, to re-circumscribe species) as an aid to taxonomy. Consequently, such studies were confined to just one or two microspecies, which had previously been recognised through traditional, authoritarian taxonomy. They have typically been based on field observations of insect–flower interactions of few populations and/or laboratory-based experimentation, most commonly emphasising the biochemistry of the pseudo-pheromone cocktails. In cases where DNA data were collected, this was usually done through early fragmentation techniques such as AFLP and microsatellites, the results being graphed with plants labelled simply according to prior microspecies identity rather than more precisely according to source population. In these features, these studies contrast strongly with the integrated analysis of an entire macrospecies Sphegodes performed by Bateman et al. [45] (Figure 18) and with the present broad-brush morphometric analysis of the entire genus.

The ethological model is best viewed as a series of steps that must occur in a particular sequence. It first requires a constant flux of variation in pseudo-pheromone cocktails. Occasionally, a novel cocktail serendipitously attracts a novel pollinator, whose subsequent interactions with the flower gradually improve its ability to repeatedly attract naïve males. Natural selection refines multiple adaptations—optimising the fit to that pollinator of pseudo-pheromone composition, flower size, three-dimensional shape, colour, markings and texture, as well as flowering time. Pre-zygotic isolation has thus been achieved, and at this point, neutral molecular markers can begin to accumulate that will, in time, become fixed within the lineage and thereby provide concrete evidence of that presumed reproductive isolation. The key question is this: At which point in this sequence of events can we be sufficiently confident that speciation has occurred—more precisely, that the presumed speciation process will not subsequently be reversed [29,45]? We will now revisit a selection of pertinent studies, partly in an attempt to answer this question but also to question whether the strength of the conclusions drawn by some previous authors exceeds the strength of the available evidence.

#### 4.6.2. Reassessing the Evidence: Do Prior Assumptions Cloud Objectivity?

We begin this reappraisal with three informative studies reporting gene-flow between *O. lupercalis* (mesospecies *Fusca*, macrospecies Fusca) and other microspecies with which it co-occurs in various regions of the Mediterranean Basin.

Stökl et al. [125] studied Mallorcan populations of *lupercalis* admixed with *O. bilunulata* (also mesospecies *Fusca*) and the later-flowering *O. fabrella* (mesospecies *Obaesa*, macrospecies Fusca), each orchid reputedly pollinated by a different species of *Andrena* bee. Ordinations of volatile compositions show partial overlap between *O. lupercalis* and *O. bilunulata*, with *O. fabrella* intermediate, but AFLP analyses suggest the presence of only two genetic entities, centred on *lupercalis* and *fabrella*. Bizarrely, plants of *bilunulata* were distributed roughly equally between the clouds formed by the other two species, rather than being intermediate. The authors of the study (p. 448) argued that “Most plants of *O. bilunulata* had the genotype of either *O. lupercalis* or *O. fabrella*. This does not mean that *O. bilunulata* does not exist as a species. It has a clearly distinguishable floral morphology and a more important floral odour, which attracts a different pollinator species than the other *Ophrys* species on Majorca. Furthermore, it is widely distributed in the Mediterranean and has the same pollinator throughout its distribution range. We therefore interpret our AFLP data as being the result of ongoing hybridization and backcrossing between the species. The similar sex pheromones mimicked by the flower and the overlapping flowering periods led to a breakdown of reproductive isolation”. But both the morphological distinctiveness and contrasting pollinators are assumed rather than rigorously demonstrated, while the absence of intermediate genotypes between the two AFLP clusters suggests the absence of primary hybrids. What evidence do we have that reproductive isolation ever existed among these taxa?

Earlier, Stökl et al. [58] had studied interaction between *O. lupercalis* and *O. iricolor* (also in macrospecies Fusca but in mesospecies *Iricolor*) on Sardinia, which showed poor separation of pseudo-pheromone cocktails and also poor separation on morphometric criteria (17 metric measurements taken from flower images). In contrast, there was stronger genetic separation based on AFLP data, but populations of both the parental species (especially *O. iricolor*) deviated from the genotypes of supposedly conspecific populations located elsewhere in the Mediterranean Basin. Furthermore, one-fifth of the plants analysed proved able to attract naïve males of the preferred pollinators of both microspecies, implying that extensive gene-flow was predictable. The conclusion drawn was that true *O. iricolor* had largely been replaced by hybrids with *O. lupercalis* in Sardinia. This in turn caused some subsequent authors to recognise Sardinian *O. iricolor* as a separate endemic species, *O. eleonorae* [126], and others to argue that the apparently widespread hybridisation between *lupercalis* and *eleonorae* had “contribut[ed] to increase in species numbers” ([25], p. 1643) in the genus. Stökl et al. [49] acknowledged the possibility that *O. iricolor* might form a genetic cline across the Mediterranean and admitted that “*Andrena*-pollinated *Ophrys* species, as [in the case of] the species of the *O. fusca* group, should tend to a high rate of nonlegitimate pollination and consequently to hybridization and introgression.” But this realisation of weak divergence in all measured parameters did not deter the authors from stating that “The high similarity of pollinator-attracting scent [among microspecies] could have been a decisive factor for the strong radiation of this group” (p. 478). But by definition, a radiation requires speciation that is concentrated in time, extensive and, most importantly, unequivocal.

Vereecken et al. [127] studied populations in southern France where *O. lupercalis* forms hybrid swarms with *O. arachnitiformis* (synonymised by some authors with *O. exaltata*)—a microspecies that belongs not only to a different mesospecies but also to a different macrospecies, Sphegodes (Figure 1 and Figure 18). Since all authors agree that the two parents represent different bona fide species, it is not surprising that their pseudo-pheromone cocktails are distinct and supposedly attract two different genera of pollinating bees. Although largely intermediate, the cocktails of the resulting hybrids also develop a minority of 23C and 25C alkenes absent from both parents. These novel compounds contribute to cocktails demonstrated to be more attractive to bee species other than those serving the parents, suggesting that new plant–pollinator relationships could arise through such hybridisation. However, the mere existence of frequent hybrids conclusively demonstrates pollinator sharing between parents whose cocktails were far more disparate. Moreover, the authors also described anarchic pollinators that varied in both the position on the labellum in which they attempted to mate with the flowers and the position(s) on the insect where the pollinaria consequently became attached. Given this valuable evidence, we view the author’s opinion that *Ophrys'* “reproductive barriers are more permeable than [previously] thought” ([127], p. 5) as seriously understated.

We now broaden our consideration of macrospecies Fusca to contemplate the supposed diversification into eight microspecies of mesospecies *Attaviria* on the island of Crete. As summarised by Baguette et al. ([25], pp. 1652–1653), “All these species are pollinated by different *Andrena* species and their flowering period is remarkably staggered from the beginning of January to the end of May. Only one of these species (*Ophrys cinereophila*) is widely distributed in Crete and in the Eastern part of the Mediterranean basin; the others are all very rare and restricted to mountain massifs in Crete. This [pattern] fits well with an incipient speciation scenario in which species differentiation based on attraction of a new pollinator within a metapopulation is followed by directional selection on flowering period leading to progressive divergence from the parent taxon. Morphological divergence has not yet taken place.” Based on our own DNA data, we doubt that molecular divergence has been any greater than morphological divergence. Contrasting pollinators plus a wide range of flowering periods would appear to be regarded as sufficient evidence to recognise several (micro)species, even though considerable altitudinal variation on Crete environmentally exaggerates the phenological spectrum, which must in any case guarantee that the identity of the preferred pollinating insect will change repeatedly through the spring. Is it not more parsimonious to assume that mesospecies *Attaviria* on Crete is represented by a morphological, phenologicial and genetic continuum, serviced by an overlapping sequence of pollinating species—in other words, that it has been artificially divided into microspecies?

But perhaps the more phylogenetically isolated macrospecies are more taxonomically tractable? Arguably the most plesiomorphic *Ophrys* macrospecies, Insectifera (Fly Orchid) was for long universally regarded as a single species, but later it was considered by Delforge [39] and others to contain three microspecies: one widespread and two geographically localised. Each putative species is said to draw its pollinators from a radically different guild of insects: wasps, bees and sawflies, respectively (the sawflies being especially casual in their behaviour; there is no preferred orientation of the fly on the flower, nor is there a preferred location on its body for pollinarium attachment) [11,128]. Sampled Fly Orchid populations demonstrably differed in the very limited sample of three morphological ‘traits’ that were recorded [59] and also differed subtly in pseudo-pheromone cocktails [129,130], but both plastid and nuclear DNA data constitute “weak but noticeable phylogeographic clustering that correlates only partially with species limits” [25]. These results were considered to “indicate a recent diversification in the three extant Fly Orchid species, which may have been further obscured by active migration and admixture across the European continent” [25,59]. But migration and admixing are exactly the processes that routinely occur *within* species; are such subtle phenotypic distinctions, poorly supported by genetic data, really sufficient to recognise multiple species?

Collectively, these valuable studies demonstrate anarchic pollinator behaviour when interacting with bee orchid flowers, contrasting radically with the adaptive perfectionism inherent in the many admiring descriptions of the extraordinary pollination process. They report supposed species that either lack genetic differentiation or possess modest genetic differentiation that contradicts prior species circumscriptions and overlapping pseudo-pheromone compositions capable of attracting multiple pollinators. Supposed morphological distinctions and pollinator preferences are tested weakly if at all. Only in the case of the hybrid swarms between *O. lupercalis* and *O. arachnitiforms* was hybrid sterility observed, and this resulted from an apparently rare case of not pre-zygotic but rather post-zygotic sterility, due to this particular population of *O. lupercalis* being tetraploid and thus incompatible with the more typically diploid *O. arachnitiformis* [127].

Yet the majority of authors of such studies fail to challenge explicitly their prior assumptions; both the microspecies circumscriptions and preferred/sole pollinator concept fed into the study survive intact in their respective discussions. We acknowledge that opinions differ among ethologists regarding the degree of pollinator specificity enjoyed by microspecies. A recent review of *Ophrys* pollination by Schatz et al. [123] usefully compared the strengths and weaknesses of contrasting methods of directly observing insects visiting *Ophrys* flowers and also offered a relatively measured and opportunistic view of pollinator specificity. Their meta-analysis suggested that the most common situation is to have a primary pollinator species but also multiple (and often taxonomically diverse) secondary pollinators.

Returning to the last of the six examples that we have chosen to highlight, we are told that “The pollinator of *O. speculum* is males of the wasp *Dasyscolia ciliata ciliata* in the western Mediterranean. In the eastern part of its range, the vicariant of *O. speculum* (*Ophrys eos*) is pollinated by the vicariant wasp *Dasyscolia ciliata araratensis*, in which females have a dark-brown body pubescence. Accordingly, the pubescence of the margins of the labellum of the vicariant *Ophrys eos* is conspicuously darker (Paulus, 2006)” ([25], p. 1647). Thus, even a subspecific difference in the identity of preferred pollinators can be cited as justification for further taxonomic division into microspecies of macrospecies Speculum. Moreover, in cases where a named *Ophrys* is shown to possess multiple pollinating species, it is immediately seen as ripe for division into multiple species, as was recently argued by Paulus [42] for *Ophrys bombyliflora* and its seven reported pollinators. From this perspective, the possession of multiple pollinators is regarded as undermining previous (often widely recognised) species circumscriptions, necessitating division into additional microspecies. Each newly minted microspecies is championed by a different pollinating insect, which in effect dictates species recognition among the orchids. Rarely has a taxonomic dog been so determinedly wagged by its own evolutionary tale [27,28,45].

#### 4.6.3. Reproductive Isolation and Lineage Separation Are Only Assumptions

All that is required in order to gain a radically different perspective on the accumulated data is to apply the principles inherent in mainstream neodarwinian microevolution and to view the evolution of *Ophrys* through the same lens as any other genus of vascular plants. First principles predict a constant flux among conspecific populations in genotype and thus in phenotype, in response to a wide range of factors: environmental, neutral and selective. Selection pressures remain capable of inducing either encouraging (directional, disruptive) or discouraging (stabilising) speciation. It is therefore expected that local populations will present at least subtly different values for mean and variance in any measured biological property. Rather, it is the *scale* of the differences among populations under comparison that dictates whether or not they are judged to be conspecific. Moreover, such comparisons should be relative rather than absolute. The expectation is that populations of different species will differ far more than populations attributed to the same species in at least some properties that are considered biologically important. Ideally, reliable discontinuities would be sought, but their emergence requires almost complete reproductive isolation, which is unlikely to be found in groups of species such as *Ophrys* that rely on leaky pre-zygotic mechanisms rooted in pollinator behaviour—a property that leaves no historical footprint and yields observations that are valid only for the present rather than the future. Thus, our demographic species concept, rooted in seeking reliable genetic and at least subtle morphometric boundaries between the same sets of populations [29,68], requires only the severe limitation of gene flow between populations rather than its complete absence.

In their exhaustive review of *Ophrys* microevolution, Baguette et al. ([25] pp. 1657–1658), argued that “Two main species concepts are in conflict: a definition based on DNA sequence homologies, and a definition based on prezygotic isolation by attraction of species-specific pollinators. According to the first definition, there are currently around ten species of bee orchids [actually nine, according to Bateman et al. [43]], whereas adoption of the prezygotic isolation criterion leads to the recognition of several hundred species.” It should be clear to any observer that our species concept is just as strongly rooted in isolation as is that employed by the ethologists. The difference is that only gene sequences (and fossils—unknown for *Ophrys*) provide historical documentation of the history of a lineage. In contrast, all of the many remaining categories of information so ably summarised by recent reviewers of *Ophrys* biology implicitly refer to just a single point in time, and most case-studies are also constrained by limited resources to very few points in space. We require the *historical evidence* of longer-term near-isolation that is provided by consistent differences in genotypes among sets of populations that are substantially greater than background levels of disparity. These differences can helpfully be visualised either as comparatively long branches in an appropriate style of tree (Figure 1) or as comparatively large distances across an appropriate style of ordination.

Baguette et al. ([25] p. 1657), then proceeded to “recommend the construction of an exhaustive and reliable molecular phylogeny of the genus *Ophrys*”, thus “providing a *definitive response* [our italics] to the endless controversy about species definition in *Ophrys*”. If only life were that simple. The repeatedly dichotomous model inherent in any attempt to build a molecular tree can be imposed on any kind of data-matrix, but it does assume that, though time, the species or populations represented by the individuals under analysis are similarly undergoing repeated dichotomies. It is the transition from dominantly reticulate relationships to a level of reproductive isolation sufficient to allow independent evolutionary fates that lies at the crux of speciation. The fundamental problem being that atemporal observations cannot provide information on whether populations (or sets of like individuals within populations) are diverging or converging. In the absence of historical data, divergence is assumed rather than demonstrated.

In the situations such as those observed *within* the nine macrospecies of *Ophrys*, wherein short molecular branches are being compared and phylogenetic resolution is consequently weak, alternative explanations for such patterns are available. For Baguette et al. [25] and other ethologists, those short branches represent a recent and active adaptive radiation, populated by hundreds of species generated on a remarkably short time-scale through strong and persistent pollinator-mediated directional and/or disruptive selection. This process reputedly leads to subtle yet evolutionarily significant changes in phenotype (most notably in the precise composition of pseudo-pheromone cocktails) that are judged (wrongly, in our view) to be sufficient in scale to constitute speciation, but nonetheless are argued to have occurred too recently for genetic signals confirming isolation to have accumulated.

However, any molecular tree based on a character-rich matrix will present such a rake-shaped topology, because all individuals of all sexually reproducing plants and animals show at least modest genetic differences. In our eyes, the phylogenetic rakes/combs evident within the macrospecies represent a range of conspecific populations (or a range of individuals sampled within a single conspecific population—both are demographic contexts in which extensive gene flow is a routine expectation). In such contexts, the sequentially dichotomous framework that is typically imposed on an evolutionary tree is an inappropriate model; an interconnected network and/or multivariate ordination would be more fitting. We conclude with regret that additional molecular phylogenies, even if much better sampled and based on next-generation sequencing, cannot offer “a definitive response to the endless controversy about species definition in *Ophrys*”.

As we have repeatedly explained in earlier publications [29,67,68], effective species circumscription requires carefully planned sampling at multiple demographic levels (individual, population and across the range of the hypothesised species) so that reciprocal illumination becomes possible between contrasting levels. The objective is to identify the optimal aggregation of sampled populations at which gene-flow is minimised. It is important to note that, especially for genera such as *Ophrys* that rarely offer any post-zygotic isolation, it is unreasonable to expect gene-flow to be non-existent among the entities recognised as species; what matters is that the genetic disparity demonstrates that gene-flow must have been severely limited, for example to a level where any incoming genes are likely to eventually be lost through drift [28,29,45]. The depression in the degree of gene flow must be sufficiently great and sufficiently prolonged to have resulted in an acceptable percentage of reliable genetic differences. If so, in most cases, those genetic differences will coincide with reliable differences in morphology and other aspects of phenotype, even though they are unlikely to be causally related. As advocated by ethologists, the case for species-level differentiation is, of course, made even stronger if contrasts in flowering time, pollinator preference and/or habitat preference are also evident, but those differences should demonstrably be both substantial and prolonged if they are to seriously impede gene flow. It is essential that prior species circumscriptions initially fed into such studies are not treated as sacrosanct but rather are tested through reciprocal illumination, both among contrasting demographic levels and among contrasting categories of data.

## 5. Conclusions

Our search for iterative evolutionary trends in *Ophrys* morphology proved at best a qualified success. Despite (or perhaps because of?) our decision to record 51 characters, the genus *Ophrys* appears to approximate a morphological continuum irrespective of the taxonomic scale at which it is analysed as a genus: microspecies, mesospecies and even some macrospecies overlap. We are not the only authors to have reached this conclusion. Pausic et al. [131] found several microspecies of macrospecies Fuciflora in the former Yugoslavia to form a morphometric continuum. Hennecke and Galanos [132] used a contrasting approach to consider morphological and phenological traits as continuous variables within the whole of macrospecies Tenthredinifera, concluding that only a single species could be recognised—the macrospecies itself.

The present results are broadly congruent with our previous broad-brush studies of barcode genetics across the genus [21] and of next-generation whole-plastid sequencing combined with morphometrics across the supposedly hyper-diverse macrospecies Sphegodes [45,133], which failed to resolve either mesospecies or microspecies (Figure 18). Moreover, there are few if any obvious evolutionary trends shared among the nine molecularly self-circumscribing macrospecies, thus giving the impression that many, and perhaps most, phenotypic features of *Ophrys* plants are free to vary independently, rather than operating under strong developmental or selective constraints. This situation is likely to favour microevolution, but it is less clear whether it would facilitate macroevolution (i.e., speciation).

If our search for evolutionary trends in *Ophrys* proved underwhelming, our search for a viable mesospecies concept can only be described as a dismal failure. A biologically valid mesospecies concept capable of circumscribing “natural” species could in theory usefully generate common ground between the two radically different taxonomic views currently available. Our ‘lumping’ classification is based on nine widespread species that can readily be distinguished using any meaningful category of biological data and that have genetic profiles demonstrating adequate levels of long-term isolation; our work implies that microevolution rarely leads to macroevolution and suggests a speciation rate typical of most other groups of extant flowering plants. Competing ‘splitters’ classifications recognise several hundred species, most of them geographically localised and many of them hybridogenic, whose circumscription variously relies on subtle differences in morphology and pseudo-pheromones, and/or assumptions of extreme pollinator specificity (either singly or in some combination). The authors of these studies tacitly assume that these properties are stable through evolutionary timescales and that microevolution often leads to macroevolution, thereby implying that the genus is currently undergoing an explosive adaptive radiation.

Does any hope remain for developing a credible mesospecies concept in the future? Relative to the demographic hierarchy, herein we effectively attempted a top-down approach, seeking but failing to find morphological discontinuities within macrospecies, just as we had already failed to find clear molecular discontinuities [45]. In theory, there exists the alternative of a ‘bottom-up’ approach that, instead of being divisive, attempts to sequentially unite the plethora of microspecies until some kind of biologically meaningful boundary is reached.

The most recent study was devised with the laudable aim of constructing in a more scientific manner mesospecies from microspecies sampled for morphometric analysis (12 metric measurements) and volatiles composition ca. 25 plants each of 12 microspecies within macrospecies Fusca, largely from populations in southern France [134]. Smaller numbers of plants were Sanger-sequenced for nrITS and introns of the (phylogenetically questionable) low-copy nuclear genes *LFY* and *BGP* in order to construct a rooted Bayesian tree. Groupings in the three data-sets were then compared, and any microspecies that could be differentiated in any one of the three datasets was judged to have been adequately circumscribed (in other words, species were allowed to be cryptic in any two of the three data categories). Unfortunately, the design of the study ignored the demographic hierarchy. Genuine circumscription requires constructing species through the concatenation of individuals from multiple sampled populations, whereas only one or at most two populations of each microspecies were sampled. In addition, the ordination method chosen—partial least-squares discriminant analysis (PLS-DA)—is a more complex model than PCA that, crucially, requires the pre-assignment of individuals to taxa and “is prone to overfitting” to prior groupings ([135], p. 1). Thus, in the absence of reciprocal illumination between populations and putative taxa and in the presence of an algorithm that must be pre-programmed with the identity of the plants being analysed and aims to emphasise the distances among those prior groupings, there is no objective circumscription process. Only when two taxa are near-identical in all measured data categories will they be united in such an analysis, so it is unsurprising that Joffard et al. [134] were only able to reduce their initial spectrum of microspecies from 12 to 10.

Future studies employing a bottom-up approach will only be valid if (a) they are conducted within a lineage benefiting from unambiguous molecular circumscription (i.e., a macrospecies); (b) feature a well-conceived sampling strategy encompassing multiple populations spanning the taxon’s full geographic extent; (c) employ an analytical arsenal that encompasses extensive morphometric (rather than more superficial, ‘trait’-based) morphology, volatiles analyses, next-generation sequencing, pollinator preference and habitat preference; and (d) analyse data using algorithms that take no account of prior groupings. Perhaps the most tractable macrospecies for a such an integrated and intensive study would be Tenthredinifera; it is relatively conspicuous and hence comparatively well-recorded, is confined geographically to the Mediterranean Basin [35] and contains a manageable number of microspecies (so far!) [39]. Admittedly, quantitative studies focusing on this macrospecies have thus far been comparatively few [90,132,136,137,138,139,140].

In our opinion, research on the genus *Ophrys* has so become heavily biased by enthusiasm for what have become widely viewed as the key elements of its predominant evolutionary mechanism, notably the hypothesised selection strengthening the relationship between sexually excited insect and sexually deceptive flower, that some lacunae are left inadequately explored. One area of research that has been surprisingly under-utilised thus far in *Ophrys* studies is functional genomics [18,23,141]. In-depth genomics/transcriptomics studies of the related Eurasian terrestrial orchid genus *Dactylorhiza* have, in contrast, provided valuable insights into the roles played in speciation by both natural selection and non-selective genetic processes such as polyploidy [92,93,94], as well as elucidating important contributions from epigenetics and ecophenotypy [142,143]. Such data would address our continuing scepticism regarding the supposed fine-tuning of the pseudo-pheromone cocktails of *Ophrys*, as we consider it highly likely that their quantitative compositions are strongly influenced by both epigenetic and ecophenotypic factors that are not amenable to intense, persistent selection. Admittedly, even acquiring such datasets proved insufficient to quell disagreements between ‘lumpers’ and ‘splitters’ addressing the taxonomy of *Dactylorhiza* [36,91]. We have long been passionate advocates for superseding the traditional herbarium taxonomy with a more integrated scientific approach that explicitly considers evolutionary mechanisms [29,68], but their inclusion is no panacea; it is clear that acquiring a diversity of datasets can itself prompt a diversity of strongly held opinions.

The suggested number of ca. 400 microspecies is not the only quantitative figure that prompts concern when considering *Ophrys*. In an impressive case of both having your cake and eating it, many ethologists argue that pre-zygotic isolation is adequately effective among microspecies, but where it is demonstrably *not* effective, hybridisation can nonetheless generate a unique phenotype that can itself form a relationship with a new dedicated pollinator and so constitute the basis of yet another new species. When viewed as a symmetrical matrix of prospective parents, the 400 microspecies formally recognised within *Ophrys* represent a theoretical maximum of 79,800 primary hybrid combinations, each a potential further microspecies. Only the extreme geographic endemism of the majority of the microspecies—their localisation often becoming a worryingly self-fulfilling criterion for their initial recognition—currently precludes the erection of an almost infinite number of microspecies. Similarly, if the genus *Ophrys* is judged through molecular phylogenetics to have originated approximately 5 myr ago [24], soon after the Mediterranean Basin flooded, an explanation is needed for why as few as nine species existed after the first 4 myr of evolution but the final ca. 1 myr yielded ca. 400 species in a radiation so rapid that even the term “explosive” becomes an understatement. Lastly, unless the plethora of microspecies originated extremely recently (e.g., post-glacially), sufficient numbers of generations should have passed in isolation to allow DNA barcoding regions to have mutated and at least a very few of those mutations in each *Ophrys* microspecies to have reached fixation through selection or drift [43].

One key question that is ignored by most ethologists because of the emphasis that they implicitly place on the present rather than the past is: What proportion of incipient species predicted by the ethological species model (which, we readily accept, emerge frequently) are rapidly reabsorbed into the ancestral genetic plexus? In other words, how many such events, if viewed through just a relatively short period of time, would be seen to be transient and reticulate rather than long-term and genuinely divergent? At what point does an emerging species cease to be ‘incipient’ [45,53,57]?

The ongoing debate regarding macrospecies versus microspecies centres on two radically different interpretations of the same body of data accumulated by the *Ophrys* industry. Despite the great volume of research now available on the genus, both sides of the species concept still ultimately rely on belief. In our case, it is the belief than the great majority of the innumerable ‘incipient species’ of *Ophrys* currently extant are routine products of mainstream microevolution that will not achieve the levels of long-term isolation that should be required for recognition as distinct species; we view the microspecies as comparable with the local variants that can be found within any bona fide species of vascular plant. In the case of the ethologists, they rely on the belief that most of the myriad ‘incipient’ species currently recognised by them, which remain in the midst of active separation from their ancestral lineages, have nonetheless already become sufficiently reproductively isolated, and have gained enough distinctive features, to be recognised as bona fide species. It is a perspective that requires sceptics such as ourselves to trust that each divergence will become more evident with time as the myriad daughter lineages continue to diverge and dichotomise (perhaps even on a human timescale) into yet more microspecies. If so, a serious challenge awaits writers of field guides to the European flora, as they struggle to summarise innumerable indistinguishable ‘species’ carved out of morphological continua.

## Figures and Tables

**Table 1:** Summary of plants subjected to morphometric analysis in the present study, organised according to nine molecularly circumscribed macrospecies.

RAD-Seq (and ITS) Groups	Number of Sampled:				
	Plants	Microspecies	Mesospecies	Localities	Countries
Insectifera (A)	14	1	1	6	3
Speculum (C)	13	2	1	12	3
Bombyliflora (D)	14	1	1	14	5
Tenthredinifera (B)	31	11	1	27	5
Fusca (E)	51	33	8	46	7
Apifera (F)	15	1	1	15	6
Umbilicata (J)	52	8 *	1 *	44	3
Fuciflora (H + I)	143	23	6	68	8
Sphegodes (G)	124	33	9	104	7
*TOTAL*	*457*	*113*	*29*		

* Two of the analysed microspecies from Cyprus placed in mesospecies *Bornmuelleri* by Delforge [39] were molecularly assigned to macrospecies *Umbilicata* by Devey et al. [21] and Bateman et al. [43].

**Table 2:** Comparison of relative degrees, as measured via convex hulls, of congruence with (a) taxonomic circumscriptions of nine microspecies and five mesospecies and (b) sampling effort for microspecies at both the levels of individual plants and populations, in the three highest planes of variation evident in the principal coordinates resulting from the morphometric analysis of macrospecies *Fuciflora* (see also Figure 16). r^2^ was assessed against negative exponential curves for nine microspecies, each represented by between five and 30 individuals.

Coordinates Plotted (and % of Total Variance Represented by Plot)	Mean ± SD (and Range) of Proportion of Group-Wide Variation (PGWV: %)		PGWV vs. Sampling Effort, Microspecies (–ve expl. r^2^)	
	Microspecies	Mesospecies	Individuals	Populations
PCo1 vs. 2 (27.1)	12.8 ± 14.1 (2.6–25.3)	40.7 ± 21.4 (20.9–66.6)	0.82	0.92
PCo1 vs. 3 (25.3)	13.6 ± 10.8 (2.1–29.1)	38.5 ± 14.3 (24.3–54.2)	0.73	0.76
PCo2 vs. 3 (22.6)	13.4 ± 11.8 (1.2–35.8)	32.7 ± 13.6 (18.6–52.3)	0.53	0.67
PCo3 vs. 4 (18.5)	12.9 ± 11.2 (1.0–27.5)	33.4 ± 12.8 (19.4–44.9)	0.72	0.83

## Data Availability

The data presented in the current study are not available within the article.

## Appendix A

**Table A1:** List of 51 morphometric characters measured from 457 individuals collectively spanning the full range of phenotypic variation in the genus *Ophrys*. Numbers of the 15 characters measured in the field are italicised; colours were matched to the RHS colour chart before conversion to CIE coordinates.

**(A) Labellum** (20 characters)
1. Maximum width
2. Maximum length (excluding appendix)
3. Depth of indentation [if present], from the maximum extent of the lateral portion of the central lobe inward to the base of the notch containing the appendix
4. Maximum length of speculum
5. Maximum width of speculum
6. Speculum position relative to stigma (scale 1–3) (grades into stigma: connected to stigma: not connected to stigma)
7. Pale zone along lower half to entire margin of speculum (scale 0–2) (absent: subdued: prominent)
8. Speculum shape (scale 1–4) (entire + U + W: I I + o o: H: single ring with radiating projections + three rings)
X. Base colour immediately below speculum
9. Colour (x)
10. Colour (y)
11. Colour (Y, %)
12. Width of pale-coloured marginal zone [if present] of labellum
13. Pilosity of central lobe margin of labellum 1 mm inside the margin and immediately above the appendix (scale 0–2) (none/negligible: short: long)
14. Pilosity of central lobe margin of labellum 1 mm inside the margin and at 45° to the vertical (scale 0–2) (none/negligible: short: long)
15. Pilosity of “shoulders”/lateral lobes of labellum 1 mm inside the margin (scale 0–2(none/negligible: short: long)
16. Appendix length [if present]
17. Appendix width [if present]
18. Length of “horns” [if present]
19. Maximum length of lateral lobes [if present] following mounting
20. Degree of curvature of labellum viewed transversely from base (scale 1–3) (±flat: gently convex: lateral lobes strongly recurved)
**(B) Lateral petals and sepals** (16 characters)
21. Length of lateral petals
22. Maximum width of lateral petals
23. Basal lateral teeth on lateral petals (scale 0–2) (absent: subdued: prominent)
X. Base colour of lateral petals
24. Colour (x)
25. Colour (y)
26. Colour (Y, %)
27. Degree of curvature of lateral petals (scale 1–5) (strongly deflexed: deflexed: ±flat: recurved: strongly recurved)
28. Length of lateral sepals
29. Maximum width of lateral sepals
X. Base colour of upper half of lateral sepals
30. Colour (x)
31. Colour (y)
32. Colour (Y, %)
33. Degree of curvature of lateral sepals (scale 1–5) (strongly deflexed: deflexed: ±flat: recurved: strongly recurved)
34. Degree of curvature of median sepal (scale 1–5) (strongly deflexed: deflexed: ±flat: recurved: strongly recurved)
35. Outline shape of median sepal (scale 1–3) (basally expanded obovate: ovate: apically expanded obovate)
36. Suffusion of dark pigment in lower half of lateral sepal (0: 1 = absent/present)
**(C) Column and ovary** (3 characters)
37. Length of ovary
38. Length of column
39. Maximum width of column
**(D) Stem and inflorescence** (5 characters)
40. Stem height
41. Stem diameter immediately above leaves
42. Inflorescence length
43. Number of flowers/buds
44. Angle subtended by labellum relative to stem (scale 1–3) (0–30° = parallel: 31–60°: 61–90° = perpendicular)
**(E) Leaves and bracts** (7 characters)
45. Number of basal (spreading) leaves
46. Number of sheathing (± upright) leaves
47. Length of longest basal leaf
48. Maximum width of longest basal leaf
49. Position of maximum with relative to position along length from base (scale 1–2) (<50%: >50%, = ovate-lanceolate: obovate leaf shapes)
50. Length of basal bract
51. Maximum width of basal bract
